# Evaluating the efficacy and safety of immune checkpoint inhibitors in first and second-line treatments for recurrent and metastatic head and neck squamous cell carcinoma: a systematic review and network meta-analysis of RCTs with a focus on PD-L1 expression

**DOI:** 10.3389/fimmu.2025.1508885

**Published:** 2025-02-13

**Authors:** Wei Chen, Qiance Wei, Tong Xiao, Jinghan Lai, Mengmeng Huang, Yueran Ma, Lili Zhang, Wenxin Xue, Shui Liu, Lichaoyue Sun, Wenshu Li, Zhijun Bu, Junge Lou, Zhaolan Liu

**Affiliations:** ^1^ Department of Pharmacy, Emergency General Hospital, Beijing, China; ^2^ Centre for Evidence-based Chinese Medicine, School of Traditional Chinese Medicine, Beijing University of Chinese Medicine, Beijing, China; ^3^ Beijing Anzhen Hospital, Capital Medical University, Beijing, China; ^4^ School of Basic Medical Sciences, Capital Medical University, Beijing, China; ^5^ Beijing Luhe Hospital, Capital Medical University, Beijing, China; ^6^ Beijing Fuxing Hospital, Capital Medical University, Beijing, China; ^7^ Pharmacy Department, Aerospace Center Hospital, Beijing, China; ^8^ Department of Pharmacy, Beijing Shijitan Hospital, Capital Medical University, Beijing, China; ^9^ Department of Ultrasound Medicine, Zhengzhou Central Hospital affiliated to Zhengzhou University, Zhengzhou, China

**Keywords:** R/M HNSCC, ICIs, efficacy, safety, network meta-analysis, PD-L1 expression

## Abstract

**Introduction:**

This study systematically reviewed and conducted a network meta-analysis to assess the efficacy and safety of first-line and second-line immunotherapy treatments for recurrent and metastatic head and neck squamous cell carcinoma (R/M HNSCC). The findings aim to provide robust evidence to guide clinical decision-making.

**Methods:**

We conducted an comprehensive literature search in PubMed, Embase, Cochrane Library, and Web of Science. The outcome measures included overall survival (OS), progression-free survival (PFS), overall response rate (ORR), and grade 3 or higher adverse events (AEs ≥3). To compare the efficacy and safety of various first-line and second-line immunotherapy regimens for R/M HNSCC with different PD-L1 expression levels, we conducted a Bayesian network meta-analysis. This study is registered in the Prospective Register of Systematic Reviews (CRD42024551711).

**Results:**

This analysis included 9 randomized controlled trials (RCTs) involving 5,946 patients and seven immunotherapy regimens. Among patients with R/M HNSCC, pembrolizumab combined with chemotherapy as a first-line treatment was the only immunotherapy regimen to show a PFS benefit compared to SOC (HR = 0.92, 95% CI: 0.77–1.10); however, the difference was not statistically significant. Meanwhile, nivolumab provided the most pronounced OS benefit (HR=0.71,95%CI:0.52-0.98). Additionally, pembrolizumab exhibited the most favorable safety profile relative to SOC (OR=0.12, 95% CI: 0.05-0.29). In second-line therapy, nivolumab outperformed SOC in multiple aspects, including OS (HR=0.68, 95% CI: 0.54-0.86), ORR (OR=0.40, 95% CI: 0.17-0.95), and grade ≥3 adverse events (OR=0.32, 95% CI: 0.19-0.54). Subgroup analysis by PD-L1 expression revealed that nivolumab, compared to SOC, conferred the greatest OS benefit (HR=0.59, 95% CI: 0.34-1.00) as a first-line therapy in patients with PD-L1 expression ≥1%, while pembrolizumab combined with chemotherapy(pem-chemo) showed the most substantial PFS benefit (HR=0.82, 95% CI: 0.67-1.00). For patients with PD-L1 expression ≥20%, pem-chemo delivered the optimal OS (HR=0.60, 95% CI: 0.44-0.81) and PFS (HR=0.73, 95% CI: 0.55-0.97) outcomes compared to SOC. Furthermore, in patients with PD-L1 expression ≥1%, nivolumab as a second-line treatment demonstrated superior OS (HR=0.55, 95% CI: 0.39-0.78) and PFS (HR=0.59, 95% CI: 0.41-0.84) compared to SOC.

**Conclusions:**

These results suggest that immunotherapy may improve survival outcomes compared to SOC for patients with R/M HNSCC, while maintaining a comparable safety profile. For patients, pembrolizumab combined with chemotherapy and nivolumab as first-line treatments may represent the most optimal options, with nivolumab also showing promise as a second-line therapy. In patients with PD-L1 expression ≥1% or ≥20%, pembrolizumab combined with chemotherapy may be the preferred first-line therapy, while nivolumab remains the most favorable second-line treatment.

**Systematic review registration:**

https://www.crd.york.ac.uk/prospero/, identifier CRD42024551711.

## 1 Introduction

Head and neck cancer ranks as the sixth most common malignancy globally, with over 891,000 new cases and more than 458,000 deaths annually, predominantly due to head and neck squamous cell carcinoma (HNSCC) (1). HNSCC arises from the mucosal epithelial cells of the oral cavity, pharynx, larynx, and sinonasal tract (2). The recurrence and metastasis of these malignant tumors significantly contribute to the high mortality rate associated with HNSCC, often leading to a poor prognosis, with a median survival of less than one year (3). Currently, the EXTREME regimen (cisplatin or carboplatin combined with 5-fluorouracil and cetuximab) is the standard first-line treatment for R/M HNSCC. Although it improves the overall response rate (ORR) and median survival, its tolerability is poor, with increased incidences of adverse reactions such as skin reactions (9%), sepsis (19%), and thrombocytopenia (11%), significantly reducing patients’ quality of life (4). After disease progression following first-line therapy, standard second-line treatments include monotherapies with methotrexate, docetaxel, or cetuximab, but the median overall survival (OS) is generally less than six months (5). Consequently, researchers have been striving to develop new therapeutic strategies to prolong patient survival.

Over the past decade, studies have demonstrated the benefits and safety of immune checkpoint inhibitors (ICIs) in various tumors (6, 7). ICIs restore T-cell activity and enhance anti-tumor immune responses by binding to protein receptors on T cells (8). The FDA’s approval of nivolumab and pembrolizumab in 2016 marked the beginning of an era of immunotherapy for R/M HNSCC patients (9, 10). As more large-scale RCTs are conducted, the landscape of first-line and second-line immunotherapy for R/M HNSCC is becoming increasingly diverse. However, the optimal immunotherapy regimen balancing efficacy and safety remains unclear. PD-L1 expression, as a biomarker, can predict which patients are more likely to respond to immunotherapy, thus optimizing the potential benefit for targeted populations (11). For R/M HNSCC patients with varying levels of PD-L1 expression, there are multiple immunotherapy options available. However, it remains unclear which first-line and second-line immunotherapy regimens provide the greatest benefit for these patients.

Most RCTs directly compare immunotherapy with standard treatments, lacking direct comparative studies among different immunotherapies (5, 12). The Bayesian approach is particularly well-suited for comparing multiple treatments in the absence of direct head-to-head trials, as it seamlessly integrates direct and indirect evidence while providing probabilistic rankings (13). Unlike frequentist methods, Bayesian credible intervals offer a more intuitive quantification of uncertainty. Additionally, the Bayesian framework supports consistency checks and sensitivity analyses, ensuring that results are both robust and clinically meaningful (14). Therefore, this study employs a Bayesian framework to indirectly compare the efficacy and safety of various immunotherapy regimens and conducts a network meta-analysis to identify the optimal first-line and second-line treatments for different patient populations based on PD-L1 expression, providing evidence-based support for clinical decision-making.

## 2 Materials and methods

This network meta-analysis (NMA) follows the guidelines set forth by the Preferred Reporting Items for Systematic Reviews and Meta-Analyses (PRISMA) extension statement for network meta-analyses (**Supplementary Table 1**) (15). Given the scarcity of direct comparative RCTs for different immunotherapy regimens, the Bayesian method is employed to predict the ranking of efficacy and safety through indirect comparisons (16). To ensure transparency, reliability, and novelty, the study protocol is registered with the Prospective Register of Systematic Reviews (CRD42024551711).

### 2.1 Data sources and search strategy

A systematic search was conducted in the PubMed, EMBASE, Cochrane Library, and Web of Science databases. The search terms included “head and neck squamous cell carcinoma”, “Squamous Cell Carcinoma of the Larynx”, “randomized clinical trial”, “immune checkpoint inhibitors”, “PD-L1 inhibitor”,”PD-1 inhibitor”, “CTLA-4 inhibitor”, “pembrolizumab”, “camrelizumab”, “nivolumab”, “ipilimumab”, “durvalumab”, “tremelimumab”, “toripalimab” and “tislelizumab”. (**Supplementary Table 2**). The search covered publications from database inception until September 1, 2024, utilizing a combination of free-text and MeSH terms, without language restrictions.

### 2.2 Selection criteria

Inclusion Criteria:

1. RCTs involving patients with R/M HNSCC confirmed by histology or cytology.
2. RCTs employing immunotherapy alone or in combination as first-line or second-line treatment regimens.
3. RCTs comparing immunotherapy alone or in combination with other treatment regimens for R/M HNSCC.
4. RCTs that report at least one of the following outcome measures:

OS, defined as the time from randomization to death from any cause; progression-free survival (PFS), defined as the time from randomization to disease progression or death from any cause; ORR, defined as the proportion of patients achieving an objective response; and grade 3 or higher AEs (AEs ≥3) as defined by the Common Terminology Criteria for Adverse Events (CTCAE) of the National Cancer Institute.

Exclusion Criteria:

1. RCTs based on different phases of the same patient cohort.
2. RCTs with unclear outcome measures.
3. Reviews or case reports.

RCTs were screened based on titles and abstracts before inclusion. All included RCTs were double-checked by two reviewers to ensure that the data were the most recently published.

### 2.3 Data extraction and quality assessment

Three researchers independently extracted data from the RCTs in accordance with the Preferred Reporting Items for Systematic Reviews and Meta-Analyses (PRISMA) guidelines. Any discrepancies were resolved through discussion with a fourth author. The data extracted from each article included the trial name, NCT number, publication journal, randomization ratio, year of publication, trial phase, tumor stage, histological type, sample size, patient age and gender distribution, racial composition, PD-L1 expression status, Eastern Cooperative Oncology Group (ECOG) performance status, and treatment regimens for both the experimental and control groups. The outcomes extracted included hazard ratios (HRs) with 95% confidence intervals (CIs) for OS and PFS, and odds ratios (ORs) with 95% CIs for ORR and AEs ≥ 3.

The quality of the included RCTs was assessed using the Cochrane Risk of Bias Tool (2.0). This tool evaluates five domains: risk of bias arising from the randomization process, risk of bias due to deviations from the intended interventions, risk of bias from missing outcome data, risk of bias in the measurement of the outcome, and risk of bias in the selection of the reported result. Each RCT was categorized into one of three risk levels: low risk, high risk, and having “some concerns.”

### 2.4 Statistical analysis

The primary outcomes are OS and PFS, while the secondary outcomes include ORR and AEs≥3. The effect size for OS and PFS is expressed as HRs with 95% CIs, and the effect size for ORR and grade 3 or higher AEs is expressed as ORs with 95% CIs.

A NMA was conducted using a Bayesian model in R software, employing the “rjags” and “gemtc” packages to evaluate the efficacy and safety of immunotherapy in first and second-line treatments for R/M HNSCC (17). A random-effect model was used, establishing three independent Markov chains, each with 20,000 burn-ins and 100,000 sample iterations, with a thinning interval for each chain. The overall ranking probability of different treatment regimens’ efficacy and safety was derived from the Markov chain iterations, and results were visualized through graphical representations. Funnel plots were created using STATA 18.0 software to assess publication bias. To verify the accuracy of indirect comparisons in the NMA, a pairwise meta-analysis based on frequentist methods was conducted comparing head-to-head studies and NMA indirect comparisons (**Supplementary Table 3**).

Additionally, Revman 5.4 software was used to perform a pairwise meta-analysis based on frequentist methods, aiming to re-evaluate the efficacy and safety of first-line or second-line immunotherapy versus chemotherapy alone in R/M HNSCC patients with and without PD-L1 expression. Heterogeneity was assessed using the Q-test and *I*² statistic, with *I*² ≤ 50% or *P* ≥ 0.1 indicating low heterogeneity, and *I*² > 50% or *P* < 0.1 indicating high heterogeneity. For studies with high heterogeneity, a random-effects model was applied; otherwise, a fixed-effect model was used. Sensitivity analyses were performed on high heterogeneity studies by sequentially excluding studies from the model and comparing heterogeneity changes before and after exclusion to ensure result reliability. If significant heterogeneity changes were observed, the sources of heterogeneity were analyzed. Subgroup analyses were conducted based on OS and PFS outcomes for different PD-L1 positive patients receiving first-line/second-line treatment, comparing the efficacy of various immunotherapy regimens versus chemotherapy. The significance level was set at α = 0.05.

## 3 Results

### 3.1 Systematic review and characteristics of the included studies

**Figure 1:**
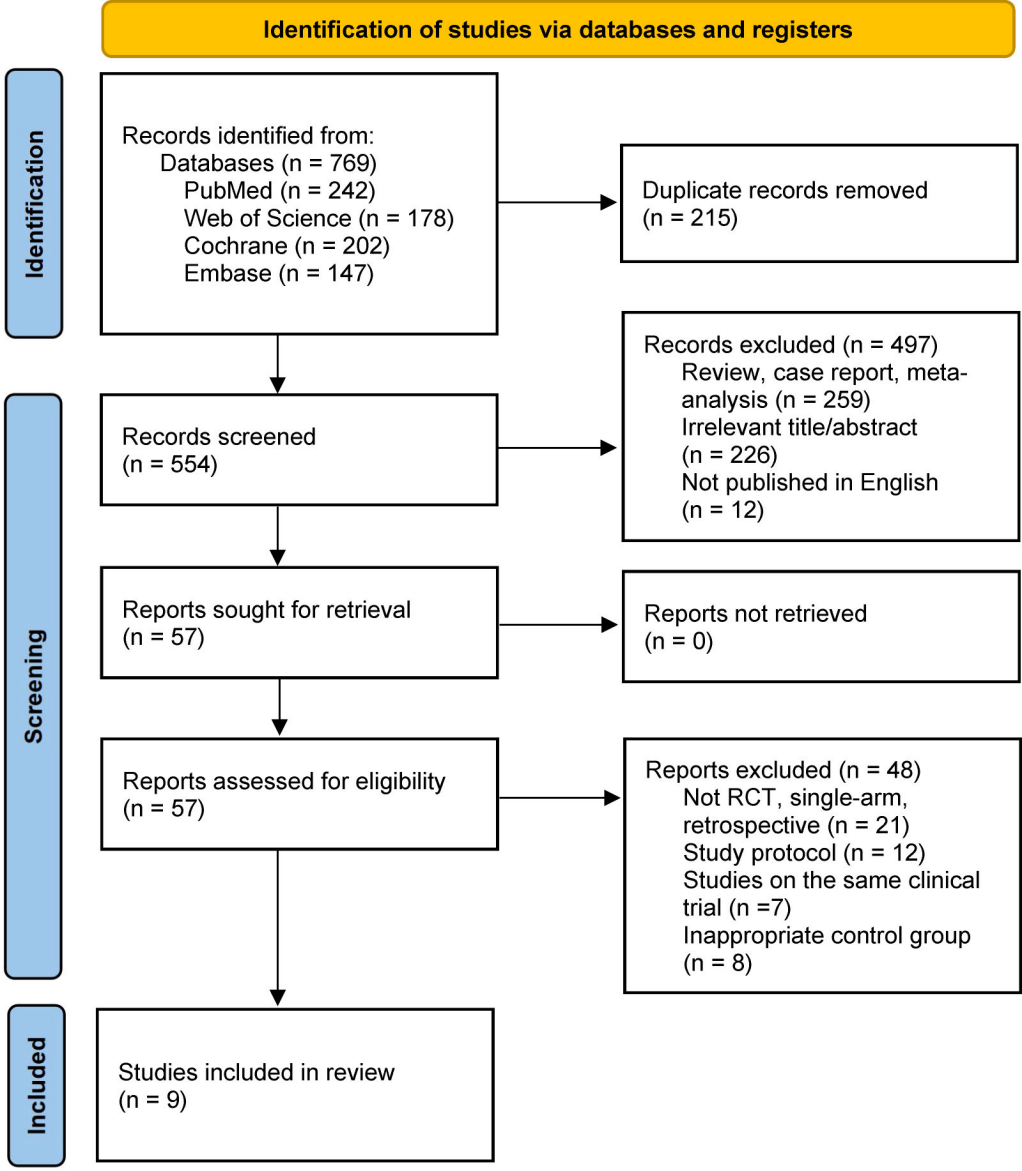
Literature search and screening flow diagram.

In the initial literature search, a total of 769 records were identified from the databases. After screening the abstracts to remove duplicates and irrelevant articles, 554 studies remained eligible for full-text review. Among these, 497 were excluded due to irrelevance (n = 226), review/meta-analyses (n = 259), or non-English publications (n = 12). Of the remaining 57 reports, 48 were excluded during eligibility assessment for reasons such as non-RCT designs (n = 21), study protocols (n = 12), duplicate clinical trials (n = 7), or inappropriate control groups (n = 8). Ultimately, 9 studies met our eligibility criteria (**Figure 1**), enrolling a total of 5,946 patients who received any of the following 8 treatments: nivolumab plus ipilimumab (nivo-ipi), durvalumab plus tremelimumab (durva-treme), durvalumab (durva), pembrolizumab (pem), pembrolizumab plus chemotherapy (pem-chemo), nivolumab (nivo), tremelimumab (treme), and standard of care (soc). Detailed information on all included studies is provided in **Tables 1**, **2**, and **Supplementary Table 4**.

**Table 1:** Baseline Characteristics of Studies Included in the Network Meta-Analysis.

Study	Source	Registered ID	Sample Size	Stage					
(Phase, Design)	(y)	(Randomization)	(Median Age/y)	(Male/ Female)	Histology	Ethnicity (%)	Intervention Arm(s)		Control Arm
CheckMate 651 (18)	JCO	NCT02741570	468/441	IV	Squamous	American (100.0)	Nivolumab 3 mg/kg Q2W	cisplatin 100 mg/m^2^ or carboplatin area under the curve 5 mg/ml/min on day 1	
(open-label, III)	2022	(1:1)	(61/62)	777/170			+ipilimumab 1 mg/kg Q6W	+5-fluorouracil 1000 mg/m²/day on days 1-4 Q3W + cetuximab 400 mg/m² on day 1, 250 mg/m² Q1W	
KESTREL (19)	ANN	NCT02551159	204/413/206	IV	Squamous	White (73.3) Asian (24.9)	Durvalumab 1500 mg Q4W	cisplatin 100 mg/m2 or carboplatin at an area under the curve of 5 mg/ml/min on day 1	
(open-label, III)	2023	(1:2:1)	(62/61/61)	689/134		Black or African American (1.3) Other (0.5)	or +tremelimumab 75 mg Q4W (for a maximum of four doses)	+5-fluorouracil 1000 mg/m²/day on days 1-4 Q3W + cetuximab 400 mg/m² on day 1, then 250 mg/m² Q1W	
KEYNOTE­048 (12)	Lancet	NCT02358031	301/281/300	IV	Squamous	Europe (31.7)	Pembrolizumab 200 mg Q3W	Cetuximab 400 mg/m², then 250 mg/m² Q1W	
(open-label, III)	2019	(1:1:1)	(62/61/61)	735/147		North America (22.3) Other (46.0)	or +carboplatin area under the curve 5 mg/m² or cisplatin 100 mg/m² +5-fluorouracil 1000 mg/m²/day 4 consecutive days Q3W	+carboplatin area under the curve 5 mg/m² or cisplatin 100 mg/m² +5-fluorouracil 1000 mg/m²/day 4 consecutive days Q3W	
KEYNOTE-040 (5)	Lancet	NCT02252042	247/248	IV	Squamous	Europe (61.6)	Pembrolizumab 200 mg Q3W	Methotrexate 40 mg/m² Q1W or docetaxel 75 mg/m² Q3W	
(open-label, III)	2018	(1:1)	(60/60)	412/83		North America (26.9) Other (11.5)		or cetuximab 250 mg/m² Q1W, then loading dose 400 mg/m²	
CheckMate 714 (20)	JAMA Oncol	NCT02823574	282/143	IV	Squamous	United Kingdom (100.0)	Nivolumab 3 mg/kg Q2W	Nivolumab 3 mg/kg Q2W	
(double-blind, II)	2023	(2:1)	(60/60)	346/79			+ipilimumab 1 mg/kg Q6W	+placebo Q2W	
EAGLE (21)	ANN Oncol	NCT02369874	240/247/249	IV	Squamous	White (80.4)	Durvalumab 10 mg/kg Q2W	Standard of care	
(open-label, III)	2020	(1:1:1)	(59/61/61)	618/118		Asian (15.4) Other (4.2)	or durvalumab 20 mg/kg Q4W +tremelimumab 1 mg/kg Q4W up to four doses, then durvalumab 10 mg/kg Q2W	(include: cetuximab, docetaxel, paclitaxel, methotrexate, 5-fluorouracil, TS-1 or capecitabine)	
CONDOR (22)	JAMA Oncol	NCT02319044	133/67/67	IV	Squamous	Canada (100.0)	Durvalumab 20 mg/kg Q4W +tremelimumab 1 mg/kg Q4W, followed by durvalumab 10 mg/kg Q2W	Tremelimumab 10 mg/kg Q4W for 7 doses then every 12 weeks for 2 additional doses	
(open-label, II)	2018	(2:1:1)	(62/62/61)	220/47			or durvalumab 10 mg/kg Q2W		
KEYNOTE-122 (23)	ANN Oncol	NCT02611960	117/116	III- IV	Squamous	North America (14.2)	Pembrolizumab 200 mg Q3W	Capecitabine 1000 mg/m² on days 1-14 Q3W	
(open-label, III)	2022	(1:1)	(51/53)	(193/40)		Asia (85.8)		or gemcitabine 1250 mg/m² on days 1-8 Q3W or docetaxel 75 mg/m² on days 1 Q3W	
CheckMate 141 (24)	Oral Oncol	NCT02105636	240/121	IV	Squamous	North America (40.2)	Nivolumab 3 mg/kg Q2W	Methotrexate 40–60 mg/m² Q1W	
(open-label, III)	2018	(2:1)	(60/61)	(61/300)		Europe (47.4) Other (12.4)		or docetaxel 30–40 mg/m² Q1W or cetuximab 400 mg/m² once then 250 mg/m² Q1W	

**Table 2:** Characteristics of included randomized controlled trials.

Study	PD-L1 Detection	PD-L1≥1% Patients (%)			PD-L1 ≥20% Patients (%)			Reported Outcomes
		Intervention(s),n(%)		Control, n (%)	Intervention(s),n(%)		Control, n (%)	
CheckMate 651	CPS	355(75.2)		372(78.3)	185(39.2)		178(37.5)	OS, PFS, ORR, grade≥3 AEs
KESTREL	TPS	/		/	63(30.9)	128(31.0)	65(31.6)	OS, PFS, ORR, grade≥3 AEs
KEYNOTE-048	CPS, TPS	257(85)	242(86)	255(85)	133(44)	126(45)	232(40.1)	OS, PFS, ORR, grade≥3 AEs
KEYNOTE-040	TPS, CPS	196(79)		191(77)	/		/	OS, PFS, ORR, grade≥3 AEs
CheckMate 714	TPS	157(55.7)		79(55.2)	/		/	OS, PFS, ORR, grade≥3 AEs
EAGLE	TPS	/		/	68(28.3)	72(29.1)	72(28.9)	OS, PFS, ORR, grade≥3 AEs
CONDOR	TPS	61(45.9)	30(44.8)	30(44.8)	/		/	OS, PFS, ORR, grade≥3 AEs
KEYNOTE-122	CPS	87(74.4)		73(62.9)	55(47.0)		46(39.7)	OS, PFS, ORR, grade≥3 AEs
CheckMate 141	TPS	88(36.7)		61(48.8)	/		/	OS, PFS, ORR, grade≥3 AEs

Quality assessment using the ROB 2.0 tool showed that 5 of the 9 included studies were evaluated as low risk, while 4 were classified as having some concerns. CheckMate 141 has certain variations in baseline patient data, leading to ‘some concerns’ regarding deviations from intended interventions. CheckMate 141, CheckMate 651, EAGLE, Keynote-048, and Keynote-040 are all open-label studies that were not double-blinded. Additionally, more than 10 patients in each study either withdrew or were lost to follow-up, thereby raising concerns regarding potential deviations from the intended interventions. Overall, all the RCTs were meticulously designed, demonstrating a high level of research quality. The specific assessment results are detailed in **Supplementary Figures 1**, **2**.

### 3.2 Pairwise meta-analysis

#### 3.2.1 Comparisons of OS, PFS, ORR

**Figure 2:**
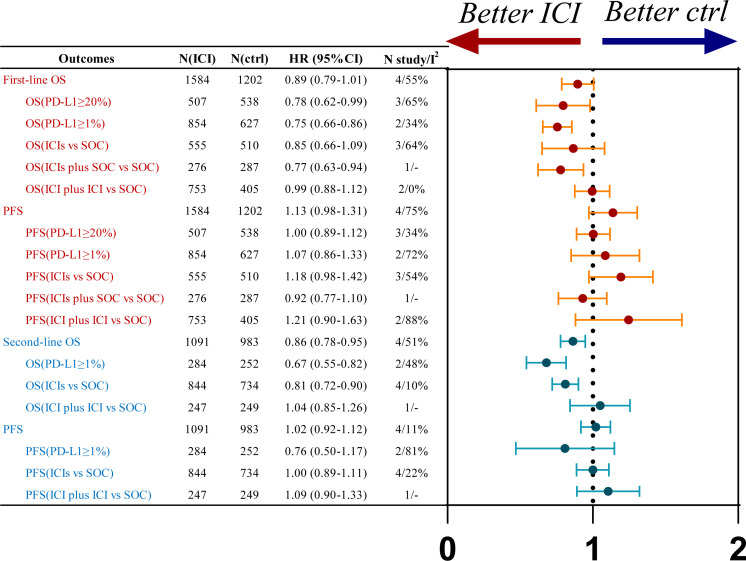
Summary forest plot of OS and PFS for first-line (Red) and second-line (Blue) treatments, ctrl, control.

Four studies investigating first-line treatment strategies reported OS, revealing moderate statistical heterogeneity (*P*=0.05, *I²*=55%). A random-effects model was utilized for the meta-analysis (**Supplementary Figure 3**). The findings indicated a trend toward improved OS in patients with R/M HNSCC who received immunotherapy, compared to the SOC (HR=0.89, 95% CI: 0.79-1.01), although the difference did not reach statistical significance. Subgroup analyses demonstrated no significant OS advantage for either ICI monotherapy (HR=0.85, 95% CI: 0.66-1.09) or dual ICI therapy (HR=0.99, 95% CI: 0.88-1.12) relative to SOC. However, significant OS benefits were observed in patients with PD-L1 expression levels ≥1% (HR=0.75, 95% CI: 0.66-0.86) and ≥20% (HR=0.78, 95% CI: 0.62-0.99) treated with immunotherapy compared to SOC (**Figure 2**; **Supplementary Figure 7**).

PFS was also assessed in the same four studies, which demonstrated significant heterogeneity (*P*=0.001, *I²*=75%). A random-effects model was applied to this analysis as well (**Supplementary Figure 4**). The results showed no significant PFS improvement in R/M HNSCC patients without PD-L1 selection who were treated with immunotherapy compared to SOC (HR=1.13, 95% CI: 0.98-1.31). Subgroup analyses further indicated no PFS benefit for ICI monotherapy (HR=1.18, 95% CI: 0.98-1.42) or dual ICI therapy (HR=1.21, 95% CI: 0.90-1.63) over SOC. Similarly, no significant PFS improvement was observed in patients with PD-L1 expression ≥1% (HR=1.07, 95% CI: 0.86-1.33) or ≥20% (HR=1.00, 95% CI: 0.89-1.12) receiving immunotherapy compared to SOC (**Figure 2**; **Supplementary Figure 8**).

**Figure 3:**
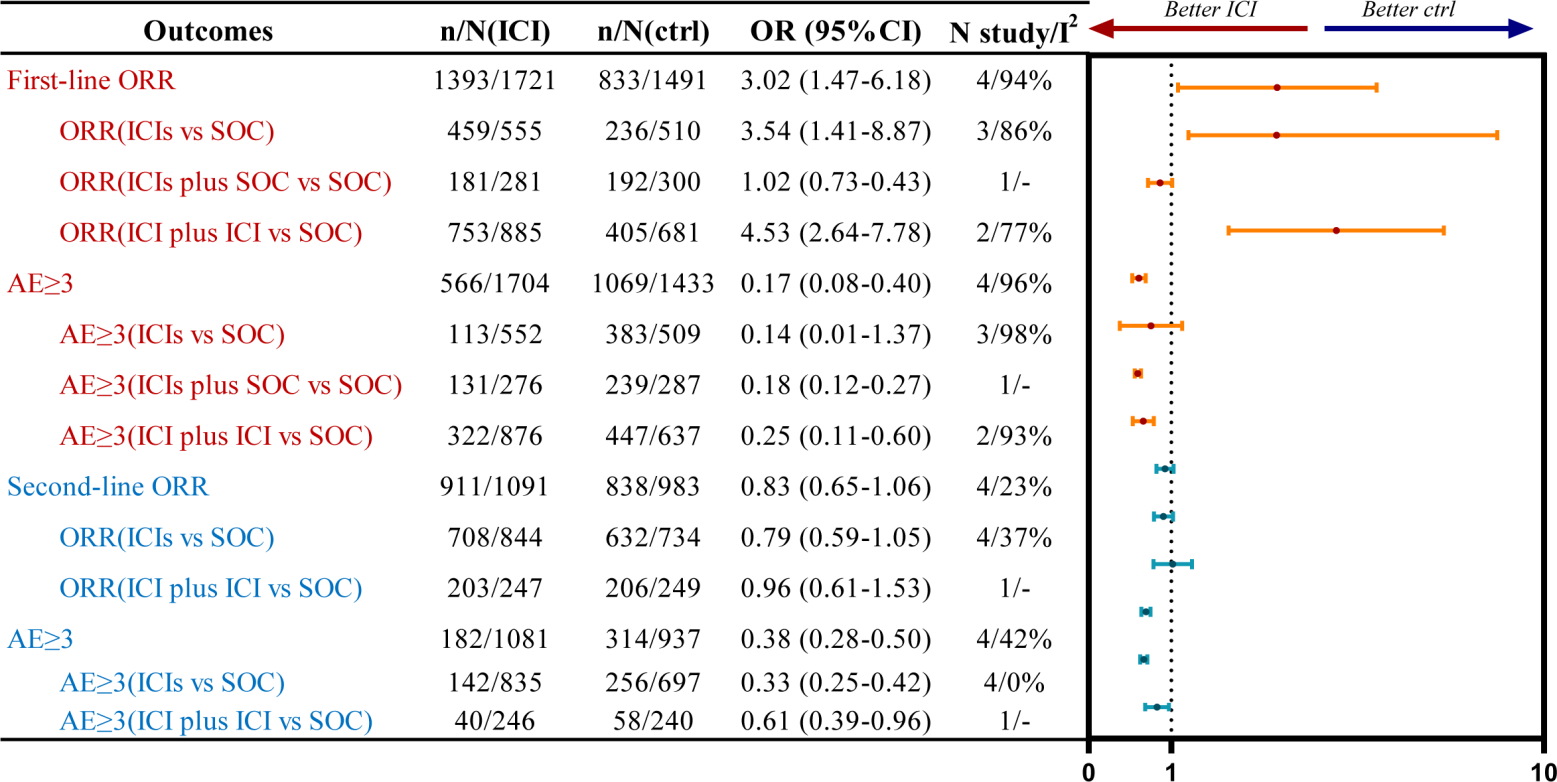
Summary forest plot of ORR and grade 3 or higher AEs for first-line (red) and second-line (blue) treatments, ctrl, control.

Four studies on first-line treatment strategies reported ORR, with significant statistical heterogeneity among the studies (*P*<0.1, *I²*=94%). A random-effects model was applied for the meta-analysis (**Figure 3**; **Supplementary Figure 5**). The results indicated that, in patients with R/M HNSCC, treatment with immunotherapy was associated with an increased ORR compared to SOC (OR=3.02, 95% CI: 1.47–6.18). Subgroup analysis revealed that both ICI monotherapy (OR=3.54, 95% CI: 1.41–8.87) and combination therapy with two ICIs (OR=4.53, 95% CI: 2.64–7.78) significantly increased the ORR compared to SOC.

Four studies on second-line treatment reported OS, with low heterogeneity (*P*=0.34, *I²*=10). A fixed-effect model was used for the meta-analysis (**Supplementary Figure 9**). The results indicated that R/M HNSCC patients (HR=0.84, 95% CI: 0.78-0.95) treated with immunotherapy had an OS benefit compared to the control group. Subgroup analysis based on PD-L1 expression showed that patients with PD-L1 expression ≥1% (HR=0.67, 95% CI: 0.55-0.82) had a significant OS benefit with immunotherapy compared to SOC. R/M HNSCC patients receiving single ICI (HR=0.81, 95% CI: 0.72-0.90) had an OS benefit over SOC, whereas dual immunotherapy and SOC had comparable efficacy (HR=1.04, 95% CI: 0.85-1.26) (**Figure 2**; **Supplementary Figure 13**).

Four studies on second-line treatment reported PFS, with low statistical heterogeneity (*P*=0.35, *I²*=11). A fixed-effect model was used for the meta-analysis (**Supplementary Figure 10**). The results indicated that R/M HNSCC patients without PD-L1 expression selection (HR=1.02, 95% CI: 0.92-1.12) had comparable PFS with immunotherapy compared to SOC. For R/M HNSCC patients with PD-L1 expression ≥1% (HR = 0.76, 95% CI: 0.50–1.17), treatment with ICIs showed a PFS benefit compared to the control group; however, the difference was not statistically significant. R/M HNSCC patients receiving single ICI (HR=1.00, 95% CI: 0.89-1.11) and dual immunotherapy (HR=1.09, 95% CI: 0.90-1.33) did not show significant PFS benefit compared to SOC (**Figure 2**; **Supplementary Figure 14**).

Four studies on second-line treatment reported ORR, with low statistical heterogeneity (*P*=0.27, *I²*=23). A fixed-effect model was used for the meta-analysis (**Figure 3**; **Supplementary Figure 11**). The results indicated no significant ORR benefit for R/M HNSCC patients (OR=0.83, 95% CI: 0.65-1.06) treated with immunotherapy compared to SOC. Subgroup analysis showed that neither single ICI (OR=0.79, 95% CI: 0.59-1.05) nor dual immunotherapy (OR=0.96, 95% CI: 0.61-1.53) provided an ORR benefit compared to SOC.

#### 3.2.2 Safety and toxicity

The incidence of AEs≥3 was used to assess the safety and toxicity of first-line and second-line ICI treatments.

Four studies evaluating first-line treatments reported the incidence of grade ≥3 AEs, with significant statistical heterogeneity observed across studies (*P*<0.1, *I²*=96%). A random-effects model was employed for the meta-analysis (**Figure 3**; **Supplementary Figure 6**). The results showed that in patients with R/M HNSCC, ICI therapy was associated with a significantly lower incidence of grade ≥3 AEs compared to the SOC (OR=0.17, 95% CI: 0.08-0.40). Subgroup analysis demonstrated that combination therapy with two ICIs (OR=0.25, 95% CI: 0.11-0.60) significantly reduced the incidence of grade ≥3 AEs compared to SOC. In contrast, ICI monotherapy (OR=0.14, 95% CI: 0.01-1.37) did not show a statistically significant safety advantage over SOC.

Four studies on second-line treatment reported the incidence of grade 3 or higher AEs, with low statistical heterogeneity (P=0.14, I²=42%). A fixed-effect model was used for the meta-analysis (**Figure 3**; **Supplementary Figure 12**). The results showed that for R/M HNSCC patients, the use of immunotherapy (OR=0.38, 95% CI: 0.28-0.50) was associated with a lower incidence of grade 3 or higher AEs compared to the control group. Subgroup analysis of intervention regimens indicated that single ICI (OR=0.33, 95% CI: 0.25-0.42) and dual immunotherapy (OR=0.61, 95% CI: 0.39-0.96) both demonstrated better safety profiles compared to SOC.

### 3.3 Network meta-analyses

#### 3.3.1 Comparisons of OS, PFS and ORR

**Figure 4:**
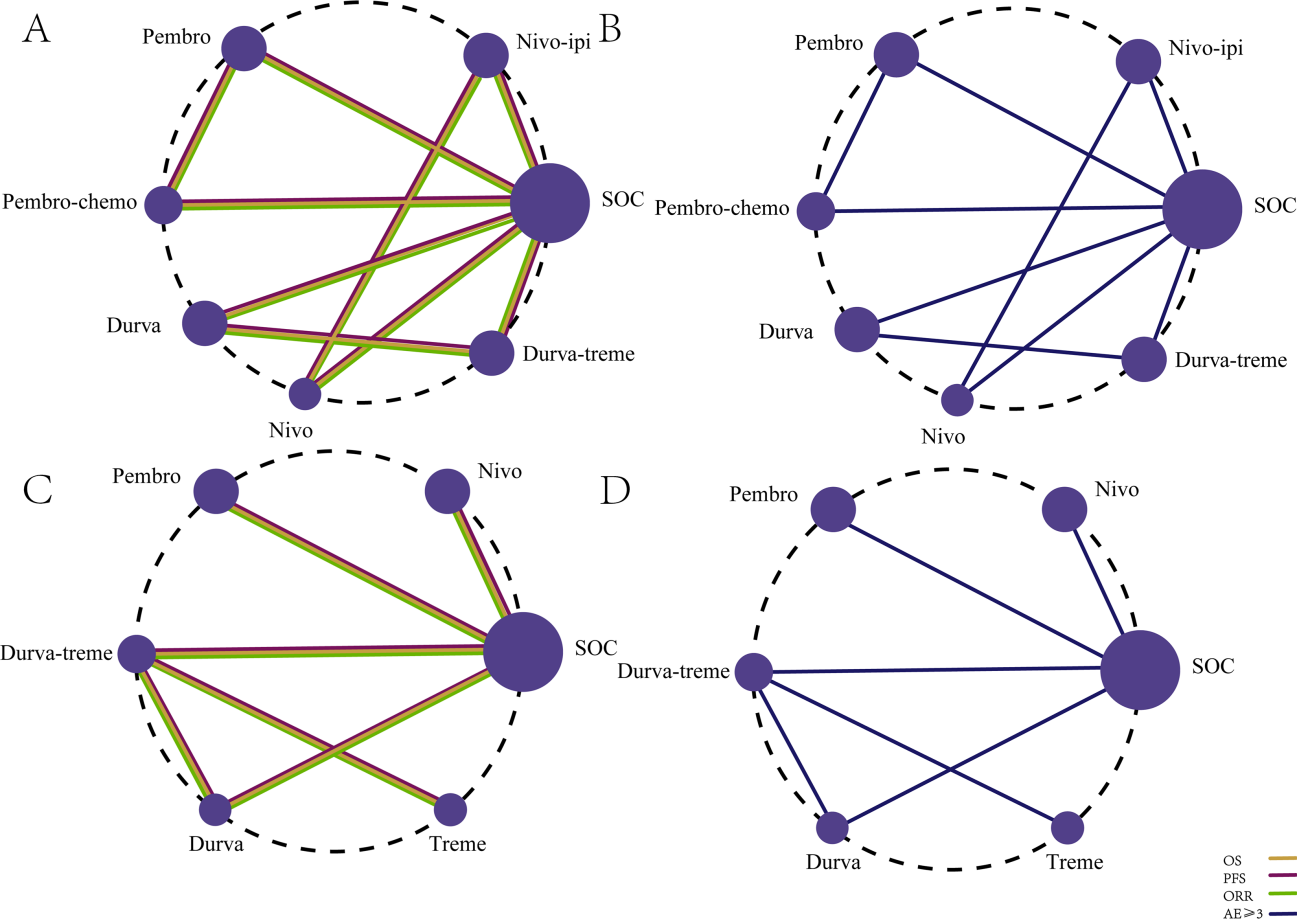
Network diagram comparing the efficacy and safety of first-line and second-line immunotherapy regimens in R/M HNSCC without PD-L1 expression selection. **(A)** OS, PFS, and ORR for first-line immunotherapy regimens. **(B)** Incidence of AEs ≥3 for first-line immunotherapy regimens. **(C)** OS, PFS, and ORR for second-line immunotherapy regimens. **(D)** Incidence of AEs ≥3 for second-line immunotherapy regimens.

The primary efficacy endpoints of this study were OS and PFS, with ORR as a secondary outcome. The NMA included seven first-line immunotherapy regimens (**Figure 4A**) and six second-line immunotherapy regimens (**Figure 4C**) for patients with R/M HNSCC.

**Figure 5:**
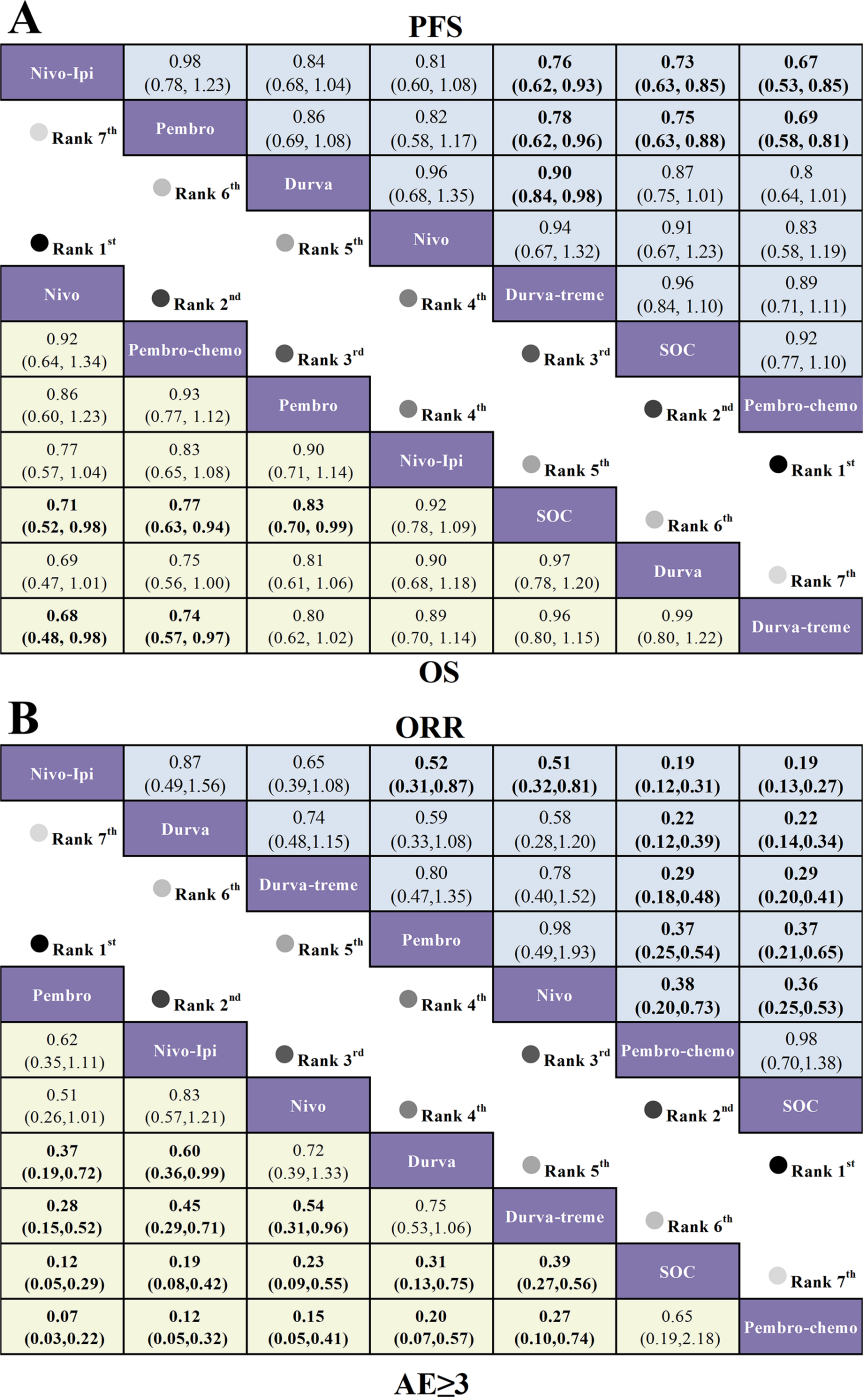
League table based on Bayesian network meta-analysis comparing the efficacy and safety of first-line immunotherapy in R/M HNSCC patients. **(A)** HR and 95% CI for OS (yellow lower triangle) and PFS (blue upper triangle), with HR < 1.00 indicating a better survival benefit. **(B)** OR and 95% CI for AEs ≥3 and ORR, with OR < 1.00 indicating a better benefit.

**Figure 6:**
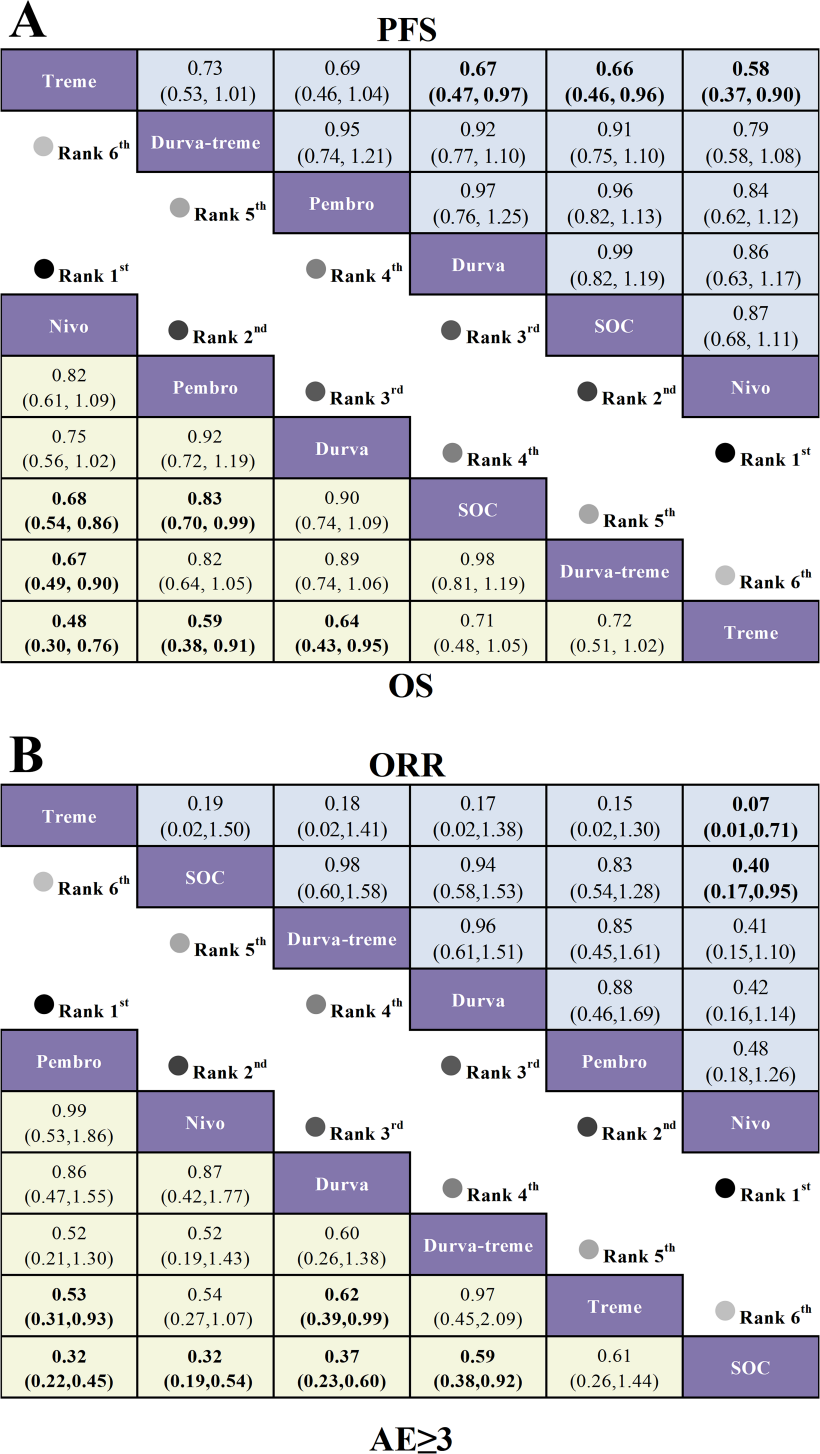
League table based on Bayesian network meta-analysis comparing the efficacy and safety of second-line immunotherapy in R/M HNSCC patients. **(A)** HR and 95% CI for OS and PFS, with HR < 1.00 indicating better survival benefit. **(B)** OR and 95% CI for AEs ≥3 and ORR, with OR < 1.00 indicating better benefit.

For OS in first-line therapy (**Figure 5A**), nivolumab (HR=0.71, 95% CI: 0.52-0.98), pem-chemo (HR=0.77, 95% CI: 0.63-0.94), and pembrolizumab monotherapy (HR=0.83, 95% CI: 0.70-0.99) demonstrated significant OS benefits compared to the standard of care (SOC). In second-line therapy (**Figure 6A**), only nivolumab (HR=0.68, 95% CI: 0.54-0.86) and pembrolizumab (HR=0.83, 95% CI: 0.70-0.99) exhibited significant OS improvements over SOC.

For PFS in first-line therapy (**Figure 5A**), nivo-ipi showed the poorest PFS outcomes across all treatment regimens. None of the immunotherapy regimens conferred a significant PFS benefit compared to SOC. Similarly, in second-line therapy (**Figure 6A**), no significant PFS improvements were observed for any immunotherapy regimen compared to SOC. However, nivolumab (HR=0.58, 95% CI: 0.37-0.90) provided the most notable PFS benefit when compared to tremelimumab.

With respect to ORR in first-line therapy (**Figure 5B**), none of the immunotherapy regimens offered an ORR advantage over SOC. The SOC showed a notably superior ORR compared to nivo-ipi (OR=0.19, 95% CI: 0.13-0.27). For second-line therapy (**Figure 6B**), pembrolizumab (OR=0.32, 95% CI: 0.22-0.45), nivolumab (OR=0.32, 95% CI: 0.19-0.54), and durvalumab (OR=0.37, 95% CI: 0.23-0.60) demonstrated significant ORR advantages over SOC.

#### 3.3.2 Safety and toxicity

Safety and toxicity were evaluated based on the incidence of grade 3 or higher AEs. The NMA included nine first-line ICI regimens for grade ≥3 AEs (**Figure 4B**) and five second-line ICI regimens (**Figure 4D**).

In first-line therapy (**Figure 5B**), all ICI monotherapies significantly reduced the incidence of grade ≥3 AEs compared to the SOC. The most notable safety benefits were observed with pembrolizumab (OR=0.12, 95% CI: 0.05-0.29), nivo-ipi (OR=0.19, 95% CI: 0.08-0.42), and nivolumab monotherapy (OR=0.19, 95% CI: 0.08-0.42). For second-line therapy (**Figure 6B**), pembrolizumab (OR=0.32, 95% CI: 0.22-0.54), nivolumab (OR=0.32, 95% CI: 0.19-0.54), and durvalumab (OR=0.37, 95% CI: 0.23-0.60) demonstrated significant safety advantages over SOC.

**Figure 7:**
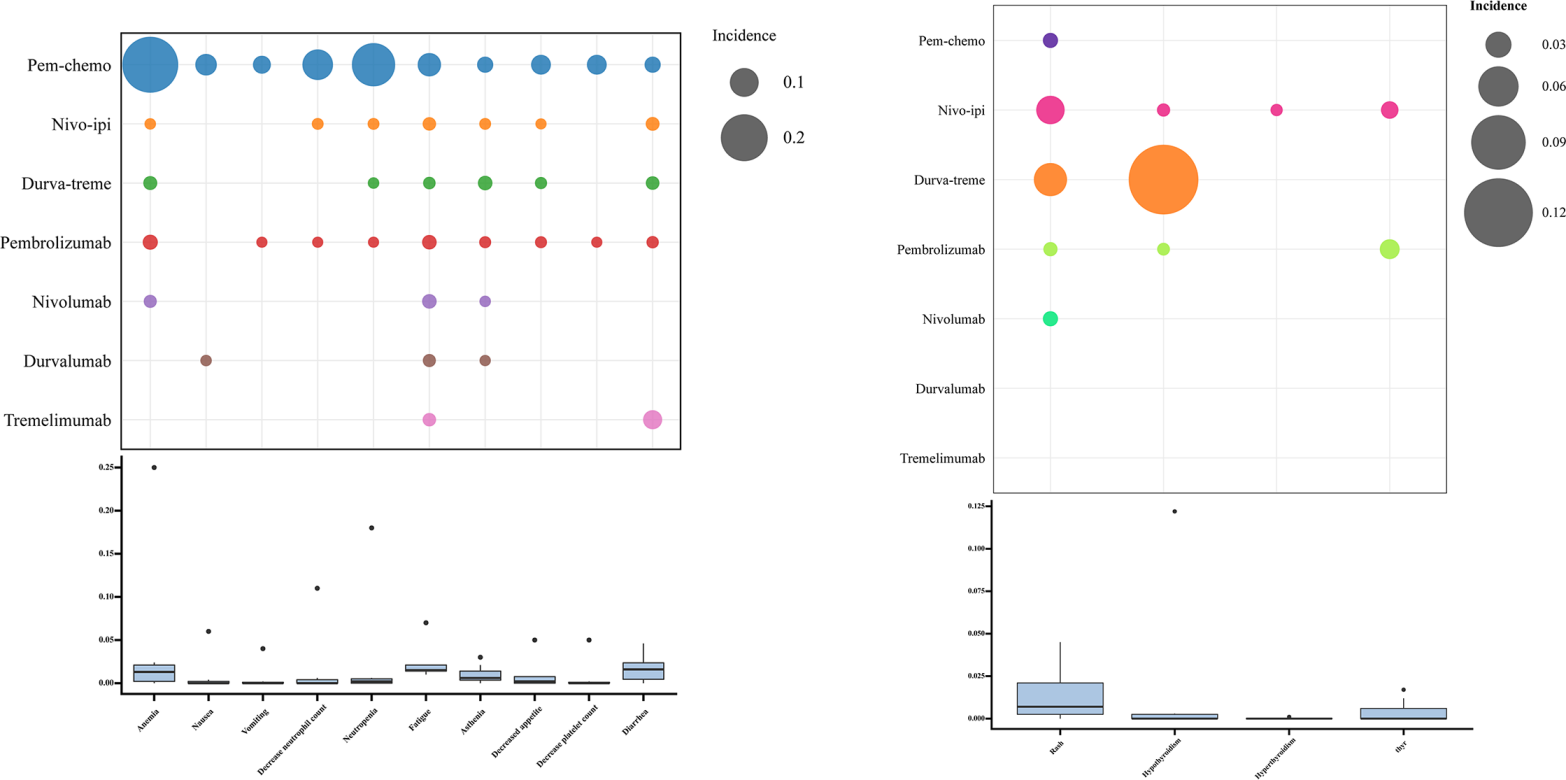
Safety Profile of Various immunotherapy Regimens. **(A)** Incidence of treatment-related Grade ≥3 adverse events. **(B)** Incidence of immune-mediated Grade ≥3 adverse events.

No new safety signals emerged during the study. The most commonly reported grade ≥3 AEs associated with immunotherapy were anemia, nausea, vomiting, decreased neutrophil count, neutropenia, fatigue, and asthenia (**Figure 7**; **Supplementary Table 5**). Frequently reported grade ≥3 immune-mediated AEs included rash, hypothyroidism, hyperthyroidism, and immune-mediated lung disease (**Supplementary Table 5**). Among all grade ≥3 AEs, pem-chemo was most likely to induce anemia, neutropenia, and decreased neutrophil count. For immune-mediated grade ≥3 AEs, durva-treme was most likely to result in rash and hypothyroidism. The incidence of treatment-related AEs, such as anemia, neutropenia, and decreased neutrophil count, varied significantly across regimens, while the spectrum of immune-mediated AEs, including hyperthyroidism and immune-mediated lung disease, was more consistent across treatments.

#### 3.3.3 Subgroup analysis

**Figure 8:**
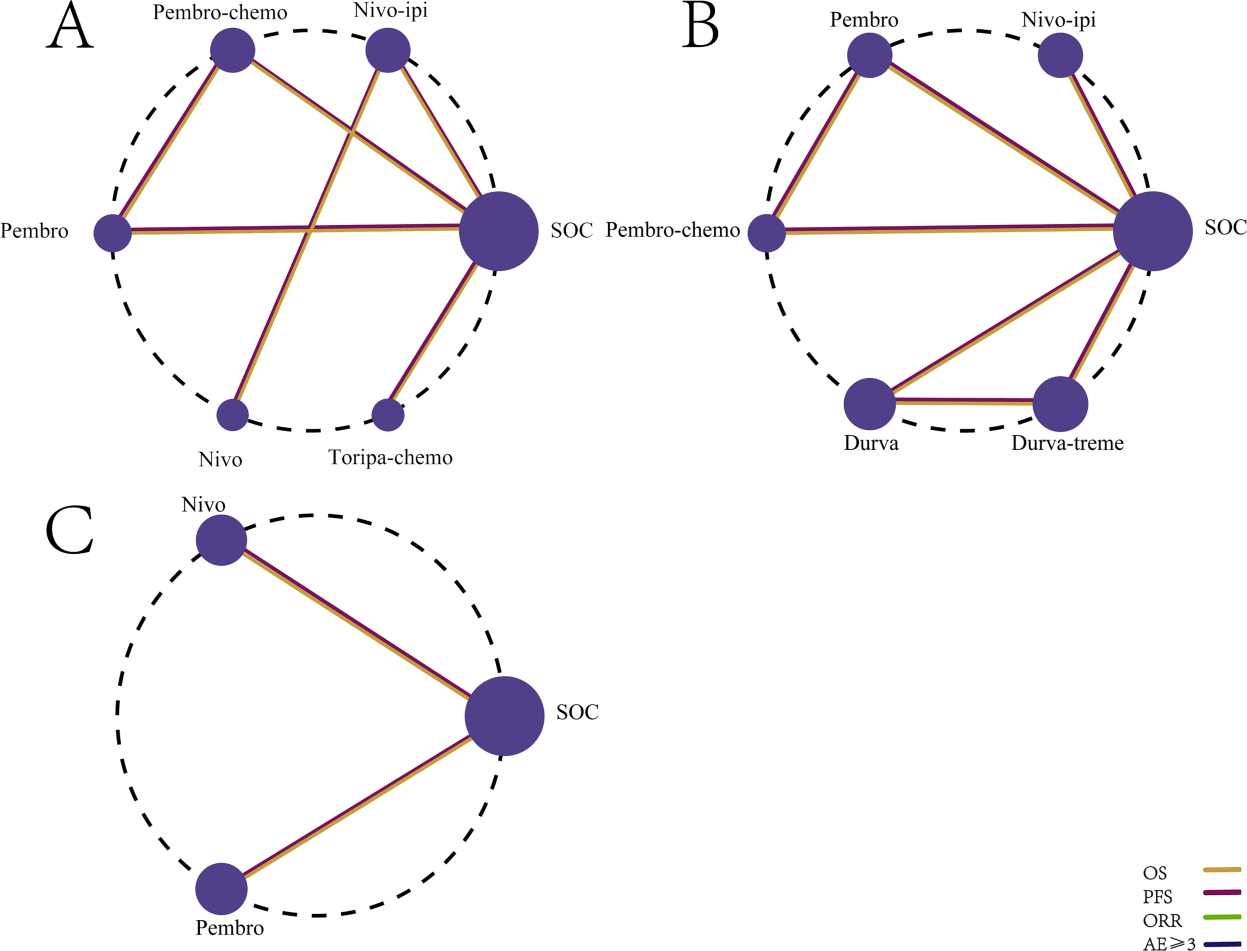
Network diagram comparing the efficacy of first-line and second-line immunotherapy regimens in R/M HNSCC with different PD-L1 expression levels. **(A)** First-line treatment regimens in patients with PD-L1 expression ≥1%. **(B)** First-line treatment regimens in patients with PD-L1 expression ≥20%. **(C)** Second-line treatment regimens in patients with PD-L1 expression ≥1%.

The NMA included six first-line immunotherapy regimens (**Figures 8A, B**) and three second-line immunotherapy regimens (**Figure 8C**) for R/M HNSCC patients with different levels of PD-L1 expression.

**Figure 9:**
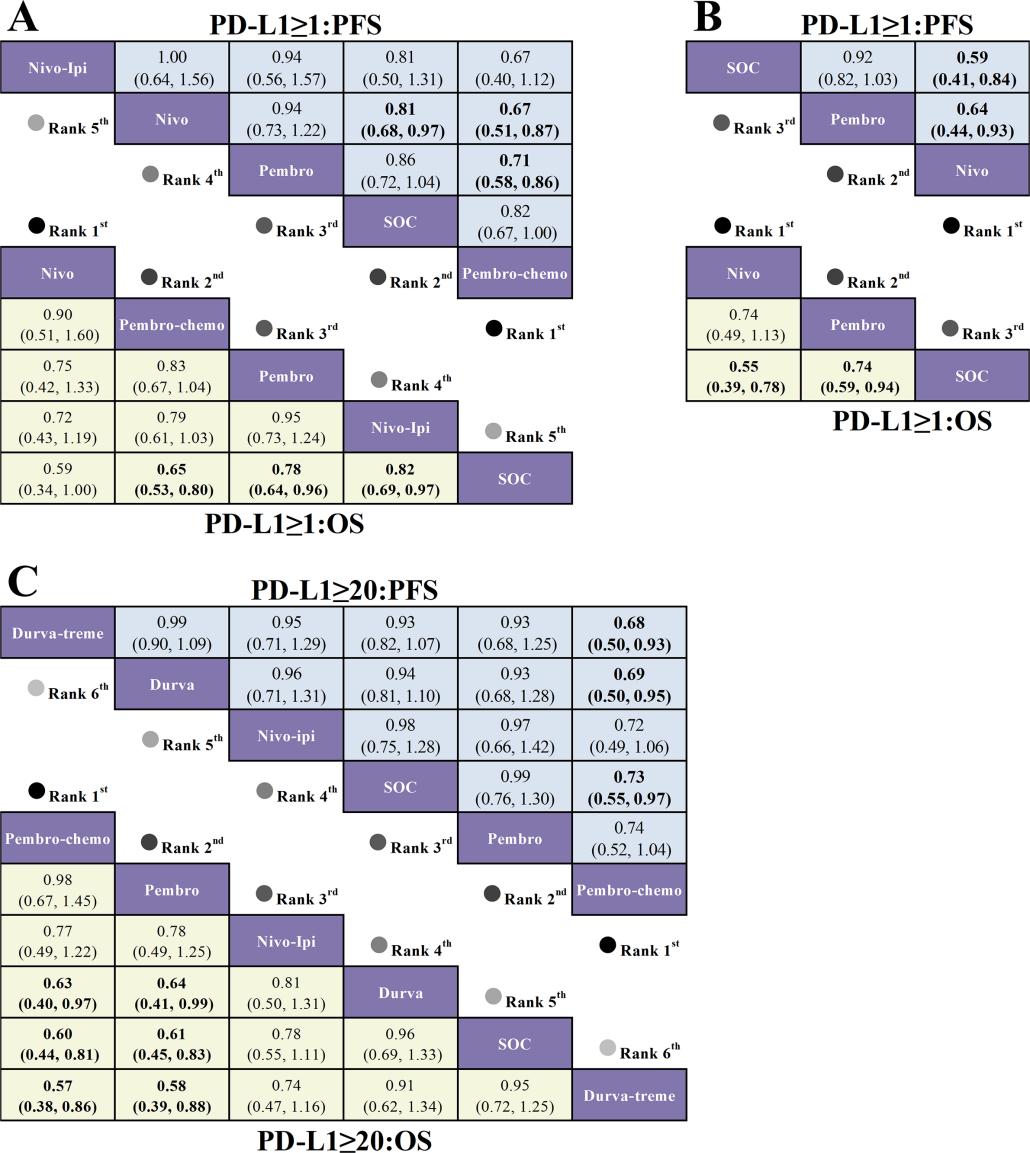
League table based on Bayesian network meta-analysis comparing the efficacy of first-line and second-line immunotherapy in R/M HNSCC patients with different PD-L1 expression levels. **(A)** HR and 95% CI for OS (yellow lower triangle) and PFS (blue upper triangle) in first-line treatment for patients with PD-L1 expression ≥1%, with HR < 1.00 indicating better survival benefit. **(B)** HR and 95% CI for OS and PFS in second-line treatment for patients with PD-L1 expression ≥1%. **(C)** HR and 95% CI for OS and PFS in first-line treatment for patients with PD-L1 expression ≥20%, with HR < 1.00 indicating better survival benefit.

For first-line treatment regarding OS (**Figures 9A, C**), R/M HNSCC patients with PD-L1 expression ≥1% showed significant OS benefits with pembrolizumab plus chemotherapy (HR=0.65, 95% CI: 0.53-0.80) and pembrolizumab (HR=0.78, 95% CI: 0.64-0.96) compared to SOC. In patients with PD-L1 expression ≥20%, both pembrolizumab plus chemotherapy (HR=0.60, 95% CI: 0.44-0.81) and pembrolizumab (HR=0.61, 95% CI: 0.45-0.83) demonstrated significant OS benefits over SOC. For second-line treatment (**Figure 9B**), patients with PD-L1 expression ≥1% had significantly prolonged OS with nivolumab (HR=0.55, 95% CI: 0.39-0.78) and pembrolizumab (HR=0.74, 95% CI: 0.58-0.94) compared to SOC.

Regarding PFS for first-line treatment (**Figures 9A, C**), in R/M HNSCC patients with PD-L1 expression ≥1%, only pembrolizumab plus chemotherapy (HR=0.82, 95% CI: 0.67-1.00) showed a statistically significant extension of PFS compared to SOC. For patients with PD-L1 expression ≥20%, only pembrolizumab plus chemotherapy (HR=0.73, 95% CI: 0.55-0.97) significantly extended PFS compared to SOC. For second-line treatment (**Figure 9B**), nivolumab (HR=0.59, 95% CI: 0.41-0.84) showed significant PFS benefits over SOC in patients with PD-L1 expression ≥1%.

### 3.4 Rank

**Figure 10:**
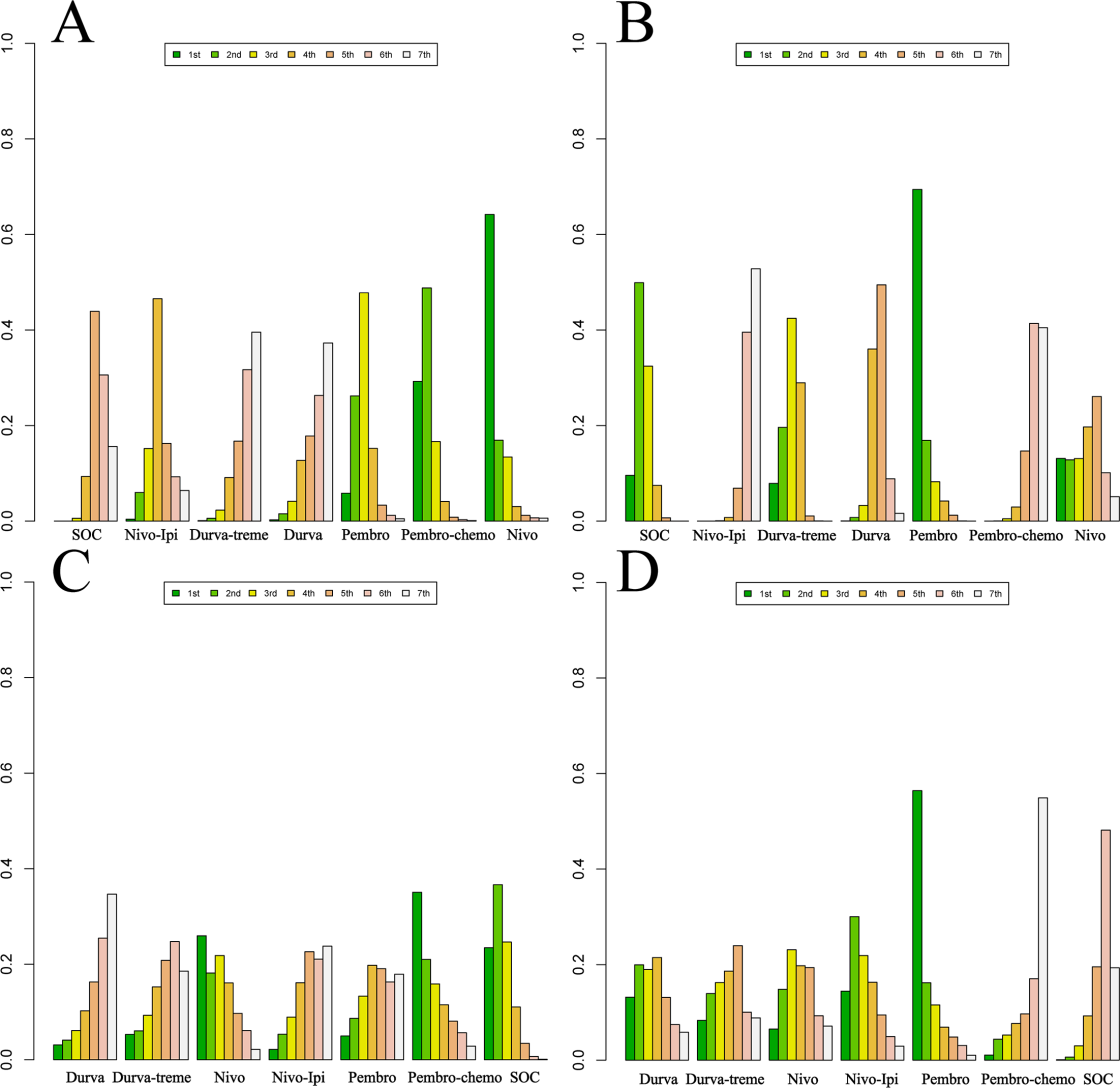
Bayesian ranking profiles for efficacy and safety of various first-line immunotherapy regimens in R/M HNSCC patients. **(A)** OS Ranking. **(B)** PFS Ranking. **(C)** ORR Ranking. **(D)** Grade ≥3 Adverse Events Ranking.

**Figure 11:**
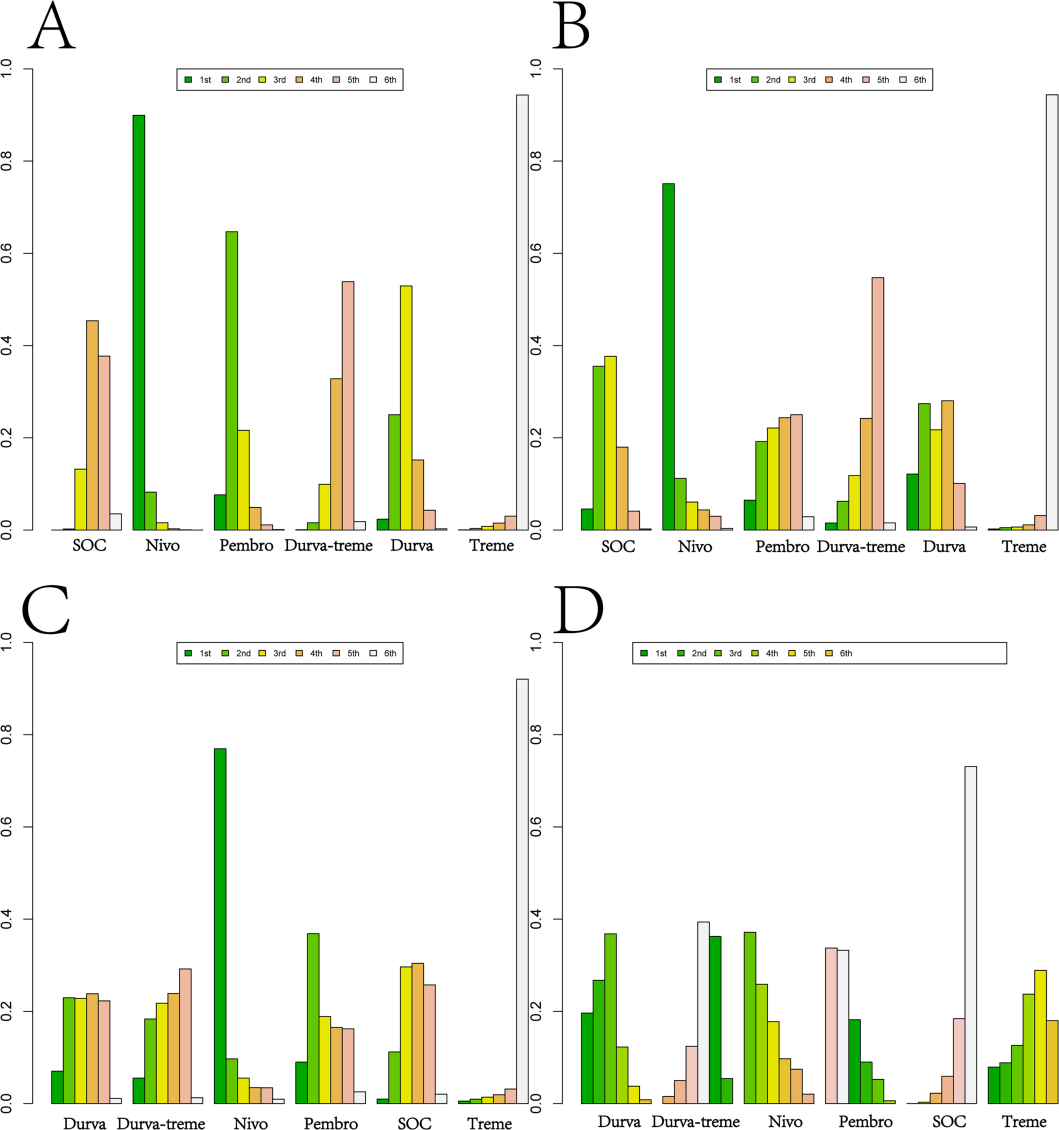
Bayesian ranking profiles for efficacy and safety of various second-line immunotherapy regimens in R/M HNSCC patients. **(A)** OS Ranking. **(B)** PFS Ranking. **(C)** ORR Ranking. **(D)** Grade ≥3 Adverse Events Ranking.

**Figure 12:**
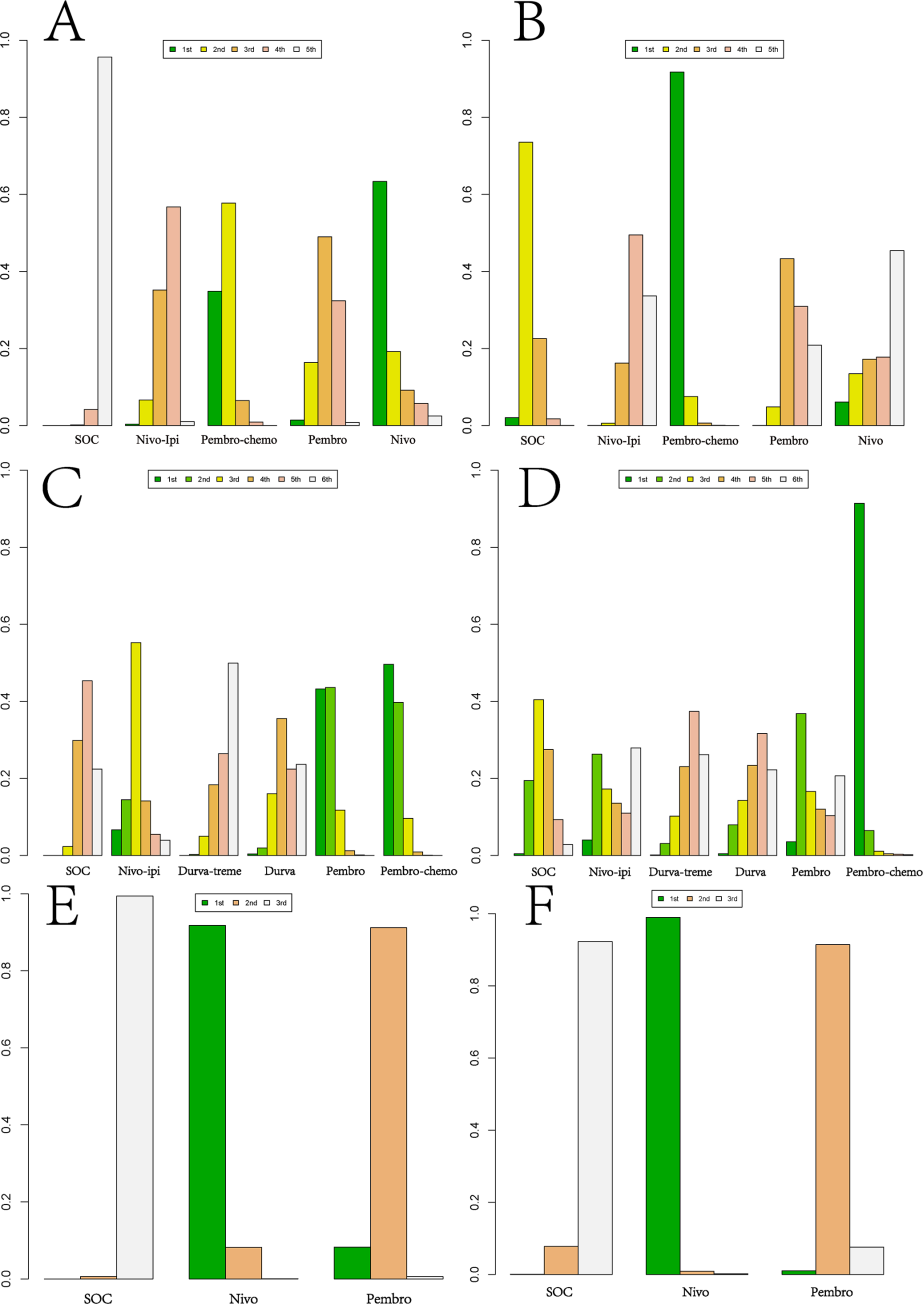
Bayesian Ranking Profiles for Efficacy and Safety of Various First-line or Second-line immunotherapy Regimens in R/M HNSCC Patients with Different PD-L1 Expression Levels. **(A)** First-line OS Ranking for PD-L1 Expression ≥1%. **(B)** First-line PFS Ranking for PD-L1 Expression ≥1%. **(C)** First-line OS Ranking for PD-L1 Expression ≥20%. **(D)** First-line PFS Ranking for PD-L1 Expression ≥20%. **(E)** Second-line OS Ranking for PD-L1 Expression ≥1%. **(F)** Second-line PFS Ranking for PD-L1 Expression ≥1%.

Ranking analysis based on Bayesian ranking profiles was conducted (**Figures 10**–**12**; **Supplementary Tables 6**–**9**). Among patients with R/M HNSCC without PD-L1 selection, nivolumab was most likely to rank first for OS with a cumulative probability of 64.17%.Pem-chemo ranked first for PFS (69.42%) and ORR (35.06%), while pembrolizumab monotherapy was most likely to rank first for grade ≥3 AEs (56.42%). Notably, pem-chemo demonstrated the best efficacy in first-line treatment, ranking first for both PFS and ORR, and second for OS.

For second-line treatment in R/M HNSCC patients, nivolumab was most likely to rank first for OS (89.91%), PFS (75.10%), and ORR (76.94%), while also ranking first for grade ≥3 AEs (37.14%). In second-line treatment, nivolumab exhibited excellent performance in both efficacy and safety, ranking first across all key outcomes: OS, PFS, ORR, and grade ≥3 AEs.

Among R/M HNSCC patients with PD-L1 expression ≥1%, nivolumab as a first-line treatment was most likely to provide the best OS benefit (63.37%), while pem-chemo was most likely to offer the greatest PFS benefit (91.76%). In second-line treatment, nivolumab demonstrated the most favorable outcomes for both OS (91.76%) and PFS (98.96%).For patients with PD-L1 expression ≥20%, pem-chemo was the most likely first-line therapy to provide the best OS (49.65%) and PFS benefit (91.42%).

### 3.5 Heterogeneity and Inconsistency

The results of the pairwise meta-analysis based on the frequentist approach are consistent with the corresponding aggregated results within the Bayesian framework (**Supplementary Table 3**). Heterogeneity was assessed using the Q test and I² statistics, indicating high heterogeneity with I² > 50% (**Figures 2**, **3**). After sequential exclusion of individual studies, the heterogeneity did not significantly decrease, suggesting the reliability of the conclusions. A funnel plot was used to analyze publication bias with OS as the outcome indicator, showing a symmetrical distribution of study points without scattered distribution, indicating a low likelihood of publication bias in this study (**Supplementary Figures 15**, **16**).

## 4 Discussion

To the best of our knowledge, this study represents the first comprehensive systematic review and network meta-analysis assessing the safety and efficacy of both first-line and second-line immunotherapies in patients with R/M HNSCC, including detailed evaluations of efficacy in subgroups with PD-L1 expression levels of ≥1% and ≥20%. Our extensive analysis yields evidence-based insights for clinical practice, summarized as follows:

1. First-line immunotherapy demonstrated a clear safety advantage compared to the SOC, but no significant efficacy benefit. In contrast, second-line immunotherapy showed significant advantages in both OS and grade ≥3 adverse events compared to SOC.
2. Combination therapy with chemotherapy and ICIs led to a marked improvement in efficacy compared to ICI monotherapy; however, this came at the cost of increased toxicity.
3. In first-line treatment for R/M HNSCC patients, pembrolizumab combined with chemotherapy provided the greatest efficacy benefit, with no statistically significant difference in safety compared to SOC, while pembrolizumab monotherapy showed the best safety profile. For patients with PD-L1 expression ≥20%, pem-chemo demonstrated the most significant efficacy benefit. Among patients with PD-L1 expression ≥1%, nivolumab offered the best OS benefit, while pem-chemo provided the greatest PFS advantage.
4. In second-line treatment, for patients, nivolumab exhibited the most outstanding performance in both efficacy and safety among all treatment options. Similarly, for patients with PD-L1 expression ≥1%, nivolumab again demonstrated the best efficacy outcomes.

Overall, immunotherapy demonstrated significant advantages in OS and Grade ≥3 adverse events compared to SOC, consistent with previous meta-analysis results (25). Given the distinct differences between first-line and second-line treatments, we analyzed them separately and performed subgroup analyses for monotherapy and combination therapy, enriching and objectifying our conclusions. Immunotherapy specifically activates the anti-tumor activity of T lymphocytes by blocking the interactions between PD-1, PD-L1, and CTLA-4, thereby enabling T cells to specifically recognize and eliminate tumor cells while sparing normal tissues (26, 27). This selective mechanism contrasts with chemotherapy, which directly destroys cancer cells but, due to its non-specific nature, may lead to increased resistance and toxicity (28). This explains the observed efficacy and safety advantages of immunotherapy over SOC. The differing mechanisms of immunotherapy and chemotherapy suggest a synergistic effect when combined, enhancing anti-tumor efficacy and explaining the significant improvement in outcomes with combined therapy (29).

This study also performed a statistical analysis of Grade ≥3 and immune-mediated adverse events, with no new safety issues identified. The incidence of severe adverse events was higher with ICI combined with chemotherapy compared to ICI monotherapy and dual immunotherapy, consistent with Dang’s meta-analysis (30). Unlike Dang’s study, our research included immune-mediated adverse events, noting a significant increase in such events with dual immunotherapy. This may be attributed to the additive effects of CTLA-4 and PD-L1/PD-1 pathways (31).

Interestingly, dual immunotherapy regimens, such as durvalumab plus tremelimumab, did not show clinical benefit over SOC in both first-line and second-line settings, and nivolumab plus ipilimumab also did not show benefit in the first-line setting compared to SOC. In contrast, ICI monotherapy demonstrated significant clinical benefits over SOC. The lack of clinical efficacy with dual immunotherapy compared to monotherapy indicates the need for further research to understand the effects of combining PD-(L)1 and CTLA-4 inhibitors in R/M HNSCC.

PD-L1 expression levels serve as biomarkers for predicting the clinical efficacy of immunotherapy in various malignancies (32). Our study found that for patients with PD-L1 expression levels of ≥20% or ≥1%, pembrolizumab combined with chemotherapy as a first-line treatment provided excellent efficacy benefits. Compared to the meta-analysis by Rodrigo et al., which only compared high PD-L1 expression(PD-L1 ≥10%) in R/M HNSCC patients using immunotherapy versus SOC, our study stratified PD-L1 expression levels and identified the optimal immunotherapy regimens (33).

This study provides a significant contribution to the existing knowledge on immunotherapy for R/M HNSCC by leveraging the Bayesian framework to yield novel insights into the comparative efficacy and safety of various regimens. Unlike prior analyses that primarily emphasized the relationship between higher PD-L1 expression and improved outcomes, this study goes beyond by stratifying PD-L1 expression levels (≥1%, ≥20%) and identifying optimal first-line and second-line treatments tailored to these subgroups. The Bayesian approach offers distinct advantages over traditional frequentist methods, including the integration of direct and indirect evidence, probabilistic treatment rankings, and robust consistency checks (34). This enables a more nuanced understanding of treatment efficacy and safety, facilitating evidence-based, personalized treatment strategies for diverse patient populations. When weighing clinical efficacy and safety, pembrolizumab combined with chemotherapy emerges as a strong first-line treatment option for R/M HNSCC patients without PD-L1 selection, while nivolumab stands out as the optimal second-line therapy. Additionally, our results demonstrate that selecting the appropriate first- or second-line immunotherapy regimen for patients with PD-L1 expression levels of ≥20% or ≥1% can lead to improved survival outcomes. These findings can complement NCCN guidelines by providing additional evidence on the most effective treatment approaches for R/M HNSCC patients based on PD-L1 expression. Future research should focus on more second-line studies combining immunotherapy with chemotherapy, such as pembrolizumab plus chemotherapy and nivolumab plus chemotherapy, to potentially expand the options for second-line treatments. Although the included studies were multicenter RCTs with patients from diverse ethnic backgrounds, we found that the majority of participants were from Europe, North America, and Asia. Less than 1% of the included patients were Black, and fewer than 3% were Hispanic. Future RCTs that include a greater number of participants from African populations or other underrepresented minority groups would enhance the comprehensiveness and generalizability of the findings. Current RCTs mainly stratify PD-L1 expression levels at 1% and 20%. Stratified analysis for patients with PD-L1 expression ≥50% in future studies could greatly aid in personalized treatment for this subgroup.

### 4.1 Limitations

Although this study draws several important conclusions, it is important to acknowledge a few limitations. First, there were variations in the SOC regimens used across the different control groups. For instance, the CheckMate 651 and KESTREL trials utilized cisplatin or carboplatin plus 5-fluorouracil in combination with cetuximab, while CheckMate 141 employed methotrexate, docetaxel, or cetuximab. While both regimens are recognized as standard first-line treatments, these differences may have introduced some degree of bias into the results. Secondly, as mentioned earlier, less than 1% of the included patients were Black, and fewer than 3% were Hispanic. The applicability of the study’s conclusions to Black individuals or other minority populations requires further consideration. Third, despite our efforts to include all relevant RCTs investigating immunotherapy for R/M HNSCC, the limited number of available trials means that some interventions were represented by only a single RCT, which may restrict the robustness of our conclusions. Fourth, as only two studies reported the safety outcomes of combination therapy compared to SOC, the finding that dual ICIs significantly reduced the incidence of grade ≥3 AEs compared to SOC should be interpreted with caution. Fifth, the included RCTs employed different methods for assessing PD-L1 expression, with some using the tumor proportion score (TPS) and others using the combined positive score (CPS). Given that CPS provides a more comprehensive reflection of the tumor microenvironment and PD-L1 expression status, it is generally preferred. However, in trials such as KESTREL or CheckMate 714, which only reported TPS, we were constrained to using TPS for PD-L1 evaluation. Finally, head and neck cancers comprise a diverse array of subtypes, such as oropharyngeal cancer, hypopharyngeal cancer, and laryngeal cancer. While survival outcomes may differ among these subtypes, the limited number of studies precludes subgroup analyses, necessitating cautious interpretation of the findings.

Despite these limitations, this study offers a thorough and comprehensive summary of randomized controlled trials on first- and second-line immunotherapy for R/M HNSCC.

## Data Availability

The original contributions presented in the study are included in the article/**Supplementary Material**. Further inquiries can be directed to the corresponding author.
