# Supplementary material for: Evaluating the efficacy and safety of immune checkpoint inhibitors in first and second-line treatments for recurrent and metastatic head and neck squamous cell carcinoma: a systematic review and network meta-analysis of RCTs with a focus on PD-L1 expression
Source: Front Immunol. 2025 Feb 13;16:1508885. doi: 10.3389/fimmu.2025.1508885 (PMC11865193; doi:10.3389/fimmu.2025.1508885)
Supplement: Supplementary file 1 [file DataSheet1.docx]

Evaluating the Efficacy and Safety of Immune Checkpoint Inhibitors in First and Second-Line Treatments for Recurrent and Metastatic Head and Neck Squamous Cell Carcinoma: A Systematic Review and Network Meta-Analysis of RCTs with a Focus on PD-L1 Expression

| **Table of Contents** | | |
| --- | --- | --- |
| Title | Content | page |
| Table S1 | PRISMA NMA Checklist of Items to Include When Reporting a Systematic Review Involving a Network Meta-analysis | 2-5 |
| Table S2 | Literature Search Strategy | 6-7 |
| Table S3 | Results of Head-to-head Meta-analysis According to Pairwise Meta-analysis and Comparisons Between Bayesian Network Meta-analysis | 8-9 |
| Table S4 | Baseline Clinical and disease Characteristics of Trials Included in the Network Meta-analysis | 10-11 |
| Table S5 | lncidence of Treatment-related Adverse Events and lmmune-mediated Adverse Events in Each lmmunotherapy Combination | 12 |
| Table S6 | Ranking Profiles in the Bayesian Network Meta-analysis（First-line） | 13-14 |
| Table S7 | Ranking Profiles in the Bayesian Network Meta-analysis(First-line/Selective expression of PD-L1) | 15 |
| Table S8 | Ranking Profiles in the Bayesian Network Meta-analysis(Second-line) | 16 |
| Table S9 | Ranking Profiles in the Bayesian Network Meta-analysis(Second-line/Selective expression of PD-L1) | 17 |
| Figure S1 | Summary of results from assessment of studies by using Cochrane Risk of Bias Tool 2.0 | 18 |
| Figure S2 | Graph of results from assessment of studies by using Cochrane Risk of Bias Tool 2.0 | 19 |
| Figure S3 | Forest Plot Comparing First-line Immunotherapy vs. SOC in Patients with R/M  HNSCC Using OS as the Endpoint | 19 |
| Figure S4 | Forest Plot Comparing First-line Immunotherapy vs. SOC in Patients with R/M  HNSCC Using PFS as the Endpoint | 20 |
| Figure S5 | Forest Plot Comparing First-line Immunotherapy vs. SOC in Patients with R/M  HNSCC Using ORR as the Endpoint | 20 |
| Figure S6 | Forest Plot Comparing First-line Immunotherapy vs. SOC in Patients with R/M  HNSCC Using AE≥3 as the Endpoint | 21 |
| Figure S7 | Forest Plot Comparing First-line Immunotherapy vs. SOC in Patients with R/M HNSCC Grouped by PD-L1 Expression Levels Using OS as the Endpoint | 21 |
| Figure S8 | Forest Plot Comparing First-line Immunotherapy vs. SOC in Patients with R/M HNSCC Grouped by PD-L1 Expression Levels Using PFS as the Endpoint | 22 |
| Figure S9 | Forest Plot Comparing Second-line Immunotherapy vs. SOC in Patients with R/M HNSCC Using OS as the Endpoint | 22 |
| Figure S10 | Forest Plot Comparing Second-line Immunotherapy vs. SOC in Patients with R/M HNSCC Using PFS as the Endpoint | 22 |
| Figure S11 | Forest Plot Comparing Second-line Immunotherapy vs. SOC in Patients with R/M HNSCC Using ORR as the Endpoint | 23 |
| Figure S12 | Forest Plot Comparing Second-line Immunotherapy vs. SOC in Patients with R/M HNSCC Using AE≥3 as the Endpoint | 23 |
| Figure S13 | Forest Plot Comparing Second-line Immunotherapy vs. SOC in Patients with R/M HNSCC and PD-L1 Expression ≥1% Using OS as the Endpoint | 23 |
| Figure S14 | Forest Plot Comparing Second-line Immunotherapy vs. SOC in Patients with R/M HNSCC and PD-L1 Expression ≥1% Using PFS as the Endpoint | 24 |
| Figure S15 | Funnel Plot of Network Meta-analysis of First-line Immunotherapy in Patients with R/M HNSCC Using OS as the Endpoint | 24 |
| Figure S16 | Figure S16. Funnel Plot of Network Meta-analysis of Second-line Immunotherapy in Patients with R/M HNSCC Using OS as the Endpoint | 25 |

Table S1 PRISMA NMA Checklist of Items to Include When Reporting a Systematic Review Involving a Network Meta-analysis

| **Section/Topic** | **Item #** | **Checklist Item** | **Reported on Page #** |
| --- | --- | --- | --- |
| **TITLE** |  |  |  |
| Title | 1 | Identify the report as a systematic review *incorporating*  *anetwork meta-analysis (or related form of meta-analysis).* | **1** |
|  |  |  |  |
| **ABSTRACT** |  |  |  |
| Structured summary | 2 | Provide a structured summary including, as applicable:  **Background:** main objectives  **Methods:** data sources; study eligibility criteria, participants, and interventions; study appraisal; and *synthesis methods, such as network meta-analysis.*  **Results:** number of studies and participants identified; summary estimates with corresponding confidence/credible intervals; *treatment rankings may also be discussed. Authors may choose to summarize pairwise comparisons against a chosen treatment included in their analyses for brevity.*  **Discussion/Conclusions:** limitations; conclusions and implications of findings.  **Other:** systematic review registration number with registry name. | 1 |
|  |  |  |  |
| **INTRODUCTION** |  |  |  |
| Rationale | 3 | Describe the rationale for the review in the context of what is already known*, including mention of why a network meta-analysis has been conducted.* | **2** |
| Objectives | 4 | Provide an explicit statement of questions being addressed, with reference to participants, interventions, comparisons, outcomes, and study design (PICOS). | 2 |
|  |  |  |  |
| **METHODS** |  |  |  |
| Protocol and registration | 5 | Indicate whether a review protocol exists and if and where it can be accessed (e.g., Web address); and, if available, provide registration information, including registration number. | 3 |
| Eligibility criteria | 6 | Specify study characteristics (e.g., PICOS, length of follow-up) and report characteristics (e.g., years considered, language, publication status) used as criteria for eligibility, giving rationale. *Clearly describe eligible treatments included in the treatment network, and note whether any have been clustered or merged into the same node (with justification).* | 3 |
| Information sources | 7 | Describe all information sources (e.g., databases with dates of coverage, contact with study authors to identify additional studies) in the search and date last searched. | 3 |
| Search | 8 | Present full electronic search strategy for at least one database, including any limits used, such that it could be repeated. | 3，Supplementary  TableS2 |
| Study selection | 9 | State the process for selecting studies (i.e., screening, eligibility, included in systematic review, and, if applicable, included in the meta-analysis). | **3，**Table 1 |
| Data collection process | 10 | Describe method of data extraction from reports (e.g., piloted forms, independently, in duplicate) and any processes for obtaining and confirming data from investigators. | 3 |
| Data items | 11 | List and define all variables for which data were sought (e.g., PICOS, funding sources) and any assumptions and simplifications made. | 3-4 |
| **Geometry of the network** | **S1** | Describe methods used to explore the geometry of the treatment network under study and potential biases related to it. This should include how the evidence base has been graphically summarized for presentation, and what characteristics were compiled and used to describe the evidence base to readers. | **4** |
| Risk of bias within individual studies | 12 | Describe methods used for assessing risk of bias of individual studies (including specification of whether this was done at the study or outcome level), and how this information is to be used in any data synthesis. | 3-4 |
| Summary measures | 13 | State the principal summary measures (e.g., risk ratio, difference in means). *Also describe the use of additional summary measures assessed, such as treatment rankings and surface under the cumulative ranking curve (SUCRA) values, as well as modified approaches used to present summary findings from meta-analyses.* | 4 |
| Planned methods of analysis | 14 | Describe the methods of handling data and combining results of studies for each network meta-analysis. This should include, but not be limited to:   - *Handling of multi-arm trials;* - *Selection of variance structure;* - *Selection of prior distributions in Bayesian analyses; and* - *Assessment of model fit.* | 4 |
| **Assessment of Inconsistency** | **S2** | Describe the statistical methods used to evaluate the agreement of direct and indirect evidence in the treatment network(s) studied. Describe efforts taken to address its presence when found. | 4 |
| Risk of bias across studies | 15 | Specify any assessment of risk of bias that may affect the cumulative evidence (e.g., publication bias, selective reporting within studies). | 4 |
| Additional analyses | 16 | Describe methods of additional analyses if done, indicating which were pre-specified. This may include, but not be limited to, the following:   - Sensitivity or subgroup analyses; - Meta-regression analyses; - *Alternative formulations of the treatment network; and* - *Use of alternative prior distributions for Bayesian analyses (if applicable).* | 4 |
| **RESULTS†** |  |  |  |
| Study selection | 17 | Give numbers of studies screened, assessed for eligibility, and included in the review, with reasons for exclusions at each stage, ideally with a flow diagram. | 4-5，Fig1 |
| **Presentation of network structure** | **S3** | Provide a network graph of the included studies to enable visualization of the geometry of the treatment network. | 13-14，Fig4-5 |
| **Summary of network geometry** | **S4** | Provide a brief overview of characteristics of the treatment network. This may include commentary on the abundance of trials and randomized patients for the different interventions and pairwise comparisons in the network, gaps of evidence in the treatment network, and potential biases reflected by the network structure. | 18-19 |
| Study characteristics | 18 | For each study, present characteristics for which data were extracted (e.g., study size, PICOS, follow-up period) and provide the citations. | 6-9,Table 1, Table 2, Supplementary Table 4 |
| Risk of bias within studies | 19 | Present data on risk of bias of each study and, if available, any outcome level assessment. | 5,Supplementary Figure S1-2 |
| Results of individual studies | 20 | For all outcomes considered (benefits or harms), present, for each study: 1) simple summary data for each intervention group, and 2) effect estimates and confidence intervals. *Modified approaches may be needed to deal with information from larger networks.* | Supplementary Table 3 |
| Synthesis of results | 21 | Present results of each meta-analysis done, including confidence/credible intervals. *In larger networks, authors may focus on comparisons versus a particular comparator (e.g. placebo or standard care), with full findings presented in an appendix. League tables and forest plots may be considered to summarize pairwise comparisons.* If additional summary measures were explored (such as treatment rankings), these should also be presented. | 11-23 |
| **Exploration for inconsistency** | **S5** | Describe results from investigations of inconsistency. This may include such information as measures of model fit to compare consistency and inconsistency models, *P* values from statistical tests, or summary of inconsistency estimates from different parts of the treatment network. | 23,Supplementary Table 3 |
| Risk of bias across studies | 22 | Present results of any assessment of risk of bias across studies for the evidence base being studied. | 23,Supplementary Figure S15-16 |
| Results of additional analyses | 23 | Give results of additional analyses, if done (e.g., sensitivity or subgroup analyses, meta-regression analyses*, alternative network geometries studied, alternative choice of prior distributions for Bayesian analyses,* and so forth). | 10-12 |
|  |  |  |  |
| **DISCUSSION** |  |  |  |
| Summary of evidence | 24 | Summarize the main findings, including the strength of evidence for each main outcome; consider their relevance to key groups (e.g., healthcare providers, users, and policy-makers). | **23-24** |
| Limitations | 25 | Discuss limitations at study and outcome level (e.g., risk of bias), and at review level (e.g., incomplete retrieval of identified research, reporting bias). *Comment on the validity of the assumptions, such as transitivity and consistency. Comment on any concerns regarding network geometry (e.g., avoidance of certain comparisons).* | 25 |
| Conclusions | 26 | Provide a general interpretation of the results in the context of other evidence, and implications for future research. | 1-2 |
|  |  |  |  |
| **FUNDING** |  |  |  |
| Funding | 27 | Describe sources of funding for the systematic review and other support (e.g., supply of data); role of funders for the systematic review. This should also include information regarding whether funding has been received from manufacturers of treatments in the network and/or whether some of the authors are content experts with professional conflicts of interest that could affect use of treatments in the network. | NA |

PICOS = population, intervention, comparators, outcomes, study design.

* Text in italics indicate S wording specific to reporting of network meta-analyses that has been added to guidance from the PRISMA statement.

† Authors may wish to plan for use of appendices to present all relevant information in full detail for items in this section.

| **Table S2.Literature Search Strategy** | |
| --- | --- |
| **Pubmed** | (((((randomized controlled trial[pt] OR controlled clinical trial[pt] OR randomized[tiab] OR placebo[tiab] OR drug therapy[sh] OR randomly[tiab] OR trial[tiab] OR groups[tiab]) NOT (animals[mh] NOT humans[mh])))) AND (("Immune Checkpoint Inhibitors"[Mesh]) OR (((((((((((((((((((((((((((((((((Checkpoint Inhibitors, Immune[Title/Abstract]) OR (Immune Checkpoint Inhibitor[Title/Abstract])) OR (Checkpoint Inhibitor, Immune[Title/Abstract])) OR (Immune Checkpoint Blockers[Title/Abstract])) OR (Checkpoint Blockers, Immune[Title/Abstract])) OR (Immune Checkpoint Blockade[Title/Abstract])) OR (Checkpoint Blockade, Immune[Title/Abstract])) OR (Immune Checkpoint Inhibition[Title/Abstract])) OR (Checkpoint Inhibition, Immune[Title/Abstract])) OR (PD-L1 Inhibitors[Title/Abstract])) OR (PD L1 Inhibitors[Title/Abstract])) OR (PD-L1 Inhibitor[Title/Abstract])) OR (PD L1 Inhibitor[Title/Abstract])) OR (Programmed Death-Ligand 1 Inhibitors[Title/Abstract])) OR (Programmed Death Ligand 1 Inhibitors[Title/Abstract])) OR (PD-1-PD-L1 Blockade[Title/Abstract])) OR (Blockade, PD-1-PD-L1[Title/Abstract])) OR (PD 1 PD L1 Blockade[Title/Abstract])) OR (CTLA-4 Inhibitors[Title/Abstract])) OR (CTLA 4 Inhibitors[Title/Abstract])) OR (CTLA-4 Inhibitor[Title/Abstract])) OR (CTLA 4 Inhibitor[Title/Abstract])) OR (Cytotoxic T-Lymphocyte-Associated Protein 4 Inhibitors[Title/Abstract])) OR (Cytotoxic T Lymphocyte Associated Protein 4 Inhibitors[Title/Abstract])) OR (Cytotoxic T-Lymphocyte-Associated Protein 4 Inhibitor[Title/Abstract])) OR (Cytotoxic T Lymphocyte Associated Protein 4 Inhibitor[Title/Abstract])) OR (PD-1 Inhibitors[Title/Abstract])) OR (PD 1 Inhibitors[Title/Abstract])) OR (PD-1 Inhibitor[Title/Abstract])) OR (Inhibitor, PD-1[Title/Abstract])) OR (PD 1 Inhibitor[Title/Abstract])) OR (Programmed Cell Death Protein 1 Inhibitor[Title/Abstract])) OR (Programmed Cell Death Protein 1 Inhibitors[Title/Abstract])))) AND (((Recurrent[Title/Abstract]) OR (Advanced[Title/Abstract]) OR (metastatic[Title/Abstract])) AND (("Squamous Cell Carcinoma of Head and Neck"[Mesh]) OR ((((((((((((((((Head and Neck Squamous Cell Carcinomas[Title/Abstract]) OR (Squamous Cell Carcinoma of the Head and Neck[Title/Abstract])) OR (Head and Neck Squamous Cell Carcinoma[Title/Abstract])) OR (HNSCC[Title/Abstract])) OR (Carcinoma, Squamous Cell of Head and Neck[Title/Abstract])) OR (Squamous Cell Carcinoma of the Larynx[Title/Abstract])) OR (Laryngeal Squamous Cell Carcinoma[Title/Abstract])) OR (Squamous Cell Carcinoma of Larynx[Title/Abstract])) OR (Squamous Cell Carcinoma of the Nasal Cavity[Title/Abstract])) OR (Oral Tongue Squamous Cell Carcinoma[Title/Abstract])) OR (Hypopharyngeal Squamous Cell Carcinoma[Title/Abstract])) OR (Oral Squamous Cell Carcinoma[Title/Abstract])) OR (Oral Cavity Squamous Cell Carcinoma[Title/Abstract])) OR (Oral Squamous Cell Carcinomas[Title/Abstract])) OR (Squamous Cell Carcinoma of the Mouth[Title/Abstract])) OR (Oropharyngeal Squamous Cell Carcinoma[Title/Abstract])))) |
| **Web of Science** | 1: ((((((((((((TS=(Squamous Cell Carcinoma of the Head and Neck)) OR TS=(Head And Neck Squamous Cell Carcinoma$)) OR TS=(HNSCC)) OR TS=(Squamous Cell Carcinoma of the Larynx)) OR TS=(Laryngeal Squamous Cell Carcinoma)) OR TS=(Squamous Cell Carcinoma of Larynx)) OR TS=(Squamous Cell Carcinoma of the Nasal Cavity)) OR TS=(Oral Tongue Squamous Cell Carcinoma)) OR TS=(Hypopharyngeal Squamous Cell Carcinoma)) OR TS=(Oral Squamous Cell Carcinoma$)) OR TS=(Oral Cavity Squamous Cell Carcinoma)) OR TS=(Squamous Cell Carcinoma of the Mouth)) OR TS=(Oropharyngeal Squamous Cell Carcinoma) and Preprint Citation Index (Exclude – Database) Results: 117222  2: ((((((((((((((TS=(Immune Checkpoint Inhibitor$)) OR TS=(Immune Checkpoint Blocker$)) OR TS=(Immune Checkpoint Blockade)) OR TS=(Immune Checkpoint Inhibition)) OR TS=(PD-L1 Inhibitor$)) OR TS=(PD L1 Inhibitor$)) OR TS=(Programmed Death-Ligand 1 Inhibitor$)) OR TS=(PD-1-PD-L1 Blockade)) OR TS=(CTLA 4 Inhibitor$)) OR TS=(CTLA-4 Inhibitor$)) OR TS=(Cytotoxic T-Lymphocyte-Associated Protein 4 Inhibitor$)) OR TS=(Cytotoxic T Lymphocyte Associated Protein 4 Inhibitor$)) OR TS=(PD 1 Inhibitor$)) OR TS=(PD-1 Inhibitor$)) OR TS=(Programmed Cell Death Protein 1 Inhibitor$) and Preprint Citation Index (Exclude – Database) Results: 105244  3: TI=(random* OR placebo* OR trial) OR AB=(random* OR placebo*) and Preprint Citation Index (Exclude – Database) Results: 2843044  4: ((TS=(Recurrent)) OR TS=(Metastatic)) OR TS=(advanced) and Preprint Citation Index (Exclude – Database) Results: 2861177  5: #1 AND #4 and Preprint Citation Index (Exclude – Database) Results: 30698  6: #2 AND #3 AND #5 and Preprint Citation Index (Exclude – Database) Results: 178 |
| **Cochrane** | #1 MeSH descriptor: [Squamous Cell Carcinoma of Head and Neck] explode all trees 694  #2 (Head And Neck Squamous Cell Carcinoma$):ti,ab,kw 3766  #3 (Squamous Cell Carcinoma of the Head and Neck):ti,ab,kw 3560  #4 (Head and Neck Squamous Cell Carcinoma):ti,ab,kw 3766  #5 (HNSCC):ti,ab,kw 978  #6 (Squamous Cell Carcinoma of the Larynx):ti,ab,kw 722  #7 (Laryngeal Squamous Cell Carcinoma):ti,ab,kw 373  #8 (Squamous Cell Carcinoma of Larynx):ti,ab,kw 737  #9 (Squamous Cell Carcinoma of the Nasal Cavity):ti,ab,kw 26  #10 (Oral Tongue Squamous Cell Carcinoma):ti,ab,kw 136  #11 (Hypopharyngeal Squamous Cell Carcinoma):ti,ab,kw 145  #12 (Oral Squamous Cell Carcinoma$):ti,ab,kw 1891  #13 (Oral Cavity Squamous Cell Carcinoma):ti,ab,kw 778  #14 (Squamous Cell Carcinoma of the Mouth):ti,ab,kw 872  #15 (Oropharyngeal Squamous Cell Carcinoma):ti,ab,kw 596  #16 #1 OR #2 OR #3 OR #4 OR #5 OR #6 OR #7 OR #8 OR #9 OR #10 OR #11 OR #12 OR #13 OR #14 OR #15 5118  #17 (Recurrent):ti,ab,kw 41324  #18 (Metastatic):ti,ab,kw 34736  #19 (advanced):ti,ab,kw 70456  #20 #17 OR #18 OR #19 127830  #21 MeSH descriptor: [Immune Checkpoint Inhibitors] explode all trees 263  #22 (Checkpoint Inhibitors, Immune):ti,ab,kw 1534  #23 (Immune Checkpoint Inhibitors):ti,ab,kw 1534  #24 (Immune Checkpoint Blockers):ti,ab,kw 22  #25 (Immune Checkpoint Blockade):ti,ab,kw 457  #26 (Immune Checkpoint Inhibition):ti,ab,kw 485  #27 (PD-L1 Inhibitor$):ti,ab,kw 990  #28 (PD L1 Inhibitor$):ti,ab,kw 1027  #29 (Programmed Death-Ligand 1 Inhibitor$):ti,ab,kw 211  #30 (Programmed Death Ligand 1 Inhibitors):ti,ab,kw 298  #31 (CTLA 4 Inhibitors):ti,ab,kw 236  #32 (CTLA-4 Inhibitors):ti,ab,kw 234  #33 (Cytotoxic T Lymphocyte Associated Protein 4 Inhibitors):ti,ab,kw 44  #34 (Cytotoxic T-Lymphocyte-Associated Protein 4 Inhibitor):ti,ab,kw 22  #35 (PD 1 Inhibitors):ti,ab,kw 3958  #36 (PD-1 Inhibitors):ti,ab,kw 950  #37 (PD 1 Inhibitor):ti,ab,kw 4736  #38 (Programmed Cell Death Protein 1 Inhibitors):ti,ab,kw 222  #39 #21 OR #22 OR #23 OR #24 OR #25 OR #26 OR #27 OR #28 OR #29 OR #30 OR #31 OR #32 OR #33 OR #34 OR #35 OR #36 OR #37 OR #38 8091  #40 #16 AND #20 AND #39 202 |
| **Embase** | #46. #16 AND #44 AND #45 147  #45. 'randomized controlled trial'/exp OR 'randomized 1,110,852  controlled trial'  #44. #17 OR #18 OR #19 OR #20 OR #21 OR #22 OR #23 OR 68,835  #24 OR #25 OR #26 OR #27 OR #28 OR #29 OR #30 OR  #31 OR #32 OR #33 OR #34 OR #35 OR #36 OR #37 OR  #38 OR #39 OR #40 OR #41 OR #42 OR #43  #43. 'pd l1 inhibitor$':ab,ti 4,091  #42. 'programmed cell death protein 1 172  inhibitor$':ab,ti  #41. 'pd 1 inhibitor':ab,ti 3,342  #40. 'inhibitor, pd-1':ab,ti 78  #39. 'pd 1 inhibitor$':ab,ti 5,562  #38. 'pd-1 inhibitor$':ab,ti 5,564  #37. 'cytotoxic t-lymphocyte-associated protein 4 44  inhibitor$':ab,ti  #36. 'ctla 4 inhibitor$':ab,ti 845  #35. 'ctla-4 inhibitor$':ab,ti 845  #34. 'pd 1 pd l1 blockade':ab,ti 1,078  #33. 'blockade, pd-1-pd-l1':ab,ti 5  #32. 'pd-1-pd-l1 blockade':ab,ti 1,078  #31. 'programmed death ligand 1 inhibitors':ab,ti 97  #30. 'programmed death-ligand 1 inhibitors':ab,ti 99  #29. 'pd-l1 inhibitor$':ab,ti 4,091  #28. 'checkpoint inhibitor$, immune':ab,ti 67  #27. 'immune checkpoint blockade':ab,ti 10,179  #26. 'immune checkpoint blockers':ab,ti 690  #25. 'checkpoint blockers, immune':ab,ti  #24. 'checkpoint blockade, immune':ab,ti 12  #23. 'immune checkpoint inhibition':ab,ti 3,242  #22. 'checkpoint inhibition, immune':ab,ti 4  #21. 'cytotoxic t lymphocyte associated protein 4 44  inhibitor$':ab,ti  #20. 'immune checkpoint inhibitor':ab,ti 11,804  #19. 'immune checkpoint inhibitors':ab,ti 32,078  #18. 'immune checkpoint blocker':ab,ti 102  #17. 'immune checkpoint inhibitor'/exp 31,330  #16. #11 AND #15 16,436  #15. #12 OR #13 OR #14 1,728,925  #14. 'recurrent':ab,ti 529,404  #13. 'advanced':ab,ti 869,473  #12. 'metastatic':ab,ti 463,598  #11. #1 OR #2 OR #3 OR #4 OR #5 OR #7 OR #8 OR #9 OR 57,624  #10  #10. 'head and neck squamous cell carcinoma':ab,ti 18,869  #9. 'squamous cell carcinoma of head and neck':ab,ti 656  #8. 'squamous cell carcinoma of the head and 5,924  neck':ab,ti  #7. 'scchn':ab,ti 2,961  #6. 'scchn (squamous cell carcinoma of the head and  neck)':ab,ti  #5. 'hnscc':ab,ti 18,194  #4. 'hn-scc':ab,ti 67  #3. 'head neck squamous cell carcinoma':ab,ti 287  #2. 'head and neck squamous cell cancer':ab,ti 1,087  #1. 'head and neck squamous cell carcinoma'/exp 44,914 |

| **TableS3. Results of Head-to-head Meta-analysis According to Pairwise Meta-analysis and Comparisons Between Bayesian Network Meta-analysis** | | | | | | |
| --- | --- | --- | --- | --- | --- | --- |
|  | **PD, L1 all** | | **PD, L1 ≥1%** | | **PD, L1 ≥20%** | |
|  | **HR for PWNA** | **HR for NMA** | **HR for PWNA** | **HR for NMA** | **HR for PWNA** | **HR for NMA** |
| **Subgroup analysis of overall survival（first-line treatment）** | | | | | | |
| Pembro-chemo vs SOC | 0.77(0.63, 0.93) | 0.77(0.63, 0.94) | 0.65(0.53, 0.80) | 0.65(0.53, 0.80) | 0.60(0.45, 0.82) | 0.60(0.44, 0.81) |
| Pembro vs SOC | 0.83(0.70, 0.99) | 0.83(0.70, 0.99) | 0.78(0.64, 0.96) | 0.78(0.64, 0.96) | 0.61(0.45, 0.83) | 0.61(0.45, 0.83) |
| Nivo-ipi vs SOC | 0.95(0.80, 1.13) | 0.92(0.78, 1.09) | 0.82(0.69, 0.97) | 0.82(0.69, 0.97) | 0.78(0.59, 1.03) | 0.78(0.55, 1.11) |
| Durva-treme vs SOC | 1.04(0.87, 1.25) | 1.03(0.83, 1.28) | / | / | 1.05(0.80, 1.39) | 1.05(0.80, 1.39) |
| Durva vs SOC | 1.03(0.83, 1.27) | 1.04(1.25, 0.87) | / | / | 0.96(0.69, 1.32) | 0.96(0.69, 1.33) |
| Nivo vs SOC | 0.71(0.52, 0.98) | 0.71(0.52, 0.98) | / | / | / | / |
| **Subgroup analysis of progression, free survival（first-line treatment）** | | | | | | |
| Pembro-chemo vs SOC | 0.92(0.77, 1.10) | 0.92(0.77, 1.10) | 0.82(0.67, 1.00) | 0.82(0.67, 1.00) | 0.73(0.55, 0.97) | 0.73(0.55, 0.97) |
| Pembro vs SOC | 1.34(1.13, 1.59) | 1.33(1.14, 1.59) | 1.16(0.96, 1.39) | 1.16(0.96, 1.39) | 0.99(0.75, 1.29) | 0.99(0.76, 1.30) |
| Nivo-ipi vs SOC | 1.41(1.21, 1.65) | 1.37(1.18, 1.59) | 1.23(1.03, 1.47) | 1.23(0.76, 2.00) | 1.02(0.78, 1.33) | 1.02(0.78, 1.33) |
| Durva-treme vs SOC | 1.04(0.91, 1.19) | 1.04(0.91, 1.19) |  | / | 1.07(0.94, 1.23) | 1.08(0.93, 1.22) |
| Durva vs SOC | 1.15(0.99, 1.33) | 1.15(0.99, 1.33) |  | / | 1.06(0.91, 1.24) | 1.06(0.91, 1.23) |
| Nivo vs SOC | 1.10(0.81, 1.49) | 1.10(0.81, 1.49) |  | / |  |  |
| **Subgroup analysis of progression, free survival（second-line treatment）** | | | | | | |
| Nivo vs SOC | 0.68(0.54, 0.86) | 0.68(0.54, 0.86) | 0.55(0.39, 0.78) | 0.55(0.39, 0.78) | / | / |
| Pembro vs SOC | 0.83(0.70, 0.99) | 0.83(0.70, 0.99) | 0.74(0.58, 0.93) | 0.74(0.59, 0.94) | / | / |
| Durva-treme vs SOC | 1.04(0.85, 1.26) | 1.02(0.84, 1.23) | / | / | / | / |
| Durva vs SOC | 0.88(0.72, 1.08) | 0.90(0.74, 1.09) | / | / | / | / |
| **Subgroup analysis of progression, free survival（second-line treatment）** | | | | | | |
| Nivo vs SOC | 0.87(0.68, 1.11) | 1.15(0.90, 1.47) | 0.59 (0.41, 0.84) | 0.59(0.41, 0.84) | / | / |
| Pembro vs SOC | 0.96(0.82, 1.13) | 0.96(0.82, 1.13) | 0.92(0.82, 1.03) | 0.92(0.82, 1.03) | / | / |
| Durva-treme vs SOC | 1.09(0.90, 1.33) | 0.91(0.75, 1.10) | / | / | / | / |
| Durva vs SOC | 1.02(0.84, 1.25) | 0.99(0.82, 1.19) | / | / | / | / |

HR, hazard ratio; PWMA, pairwise meta-analysis; NMA, network meta-analysis; Pem, pembrolizumab; Nivo, nivolumab; ipi, ipilimumab; Durva，durvalumab ；Treme，tremelimumab；SOC，standard of care；Chemo, chemotherapy.

| **Table S4. Baseline Clinical and disease Characteristics of Trials Included in the Network Meta-analysis** | | | | | | | | | | | | | | | | | | | | | | | | | |  |
| --- | --- | --- | --- | --- | --- | --- | --- | --- | --- | --- | --- | --- | --- | --- | --- | --- | --- | --- | --- | --- | --- | --- | --- | --- | --- | --- |
|  | **CheckMate 651**  **,n=947** | | **KESTREL,n=823** | | | | **KEYNOTE-048,n=882** | | | | | | | | **KEYNOTE-040,n=495** | | | | **KEYNOTE-122,**  **n=233** | | | | **EAGLE,n=736** | | |  |
|  |  |  |  |  |  |  |  |  |  |  |  |  |  |  |  |  |  |  |  |  |  |  |  |  |  |  |
|  | **IG** | **CG** | **IG1** | **IG2** | | **CG** | **IG1** | | | **IG2** | | **CG1** | | **CG2** | **IG** | | **CG** | | **IG** | | | **CG** | **IG1** | **IG2** | **CG** |  |
|  |  |  |  |  |  |  |  |  |  |  |  |  |  |  |  |  |  |  |  |  |  |  |  |  |  |  |
| **PD-L1 expression level（CPS）** | | | | | | | | | | | | | | | | | | | | | | | | | |  |
| <1 | 19.5% | 18.1% | / | / | | / | / | | | / | | / | | / | 20% | | 22% | | / | | / | | / | / | / |  |
| ≥1 | 75.2% | 78.3% | / | / | | / | 85% | | | 86% | | 85% | | 85% | 79% | | 77% | | 74.4% | | 62.9% | | / | / | / |  |
| <20 | / | / | / | / | | / | / | | | / | | / | | / | / | | / | | / | | / | | / | / | / |  |
| ≥20 | 39.2% | 37.5% | / | / | | / | 44% | | | 45% | | 44% | | 40% | / | | / | | 37.6% | | 28.4% | | / | / | / |  |
| **PD-L1 expression level（TPS）** | | | | | | | | | | | | | | | | | | | | | | | | | |  |
| <1% | / | / | / | / | | / | / | | | / | | / | | / | / | | / | | / | | / | | / | / | / |  |
| ≥1% | / | / | / | / | | / | / | | | / | | / | | / | / | | / | | / | | / | | / | / | / |  |
| <20% | / | / | 69.1% | 69.0% | | 68.4% | / | | | / | | / | | / | / | | / | | / | | / | | 71.7% | 70.9% | 71.1% |  |
| ≥20% | / | / | 30.9% | 31.0% | | 31.6% | / | | | / | | / | | / | / | | / | | / | | / | | 28.3% | 29.1% | 28.9% |  |
| **Gender** | | | | | | | | | | | | | | | | | | | | | | | | | |  |
| Male | 80.5% | 83.6% | 85.8% | 82.3% | | 84.5% | 83% | | | 20% | | 13% | | 17% | 84% | | 83% | | 83.8% | | 81.9% | | 84.2% | 84.6% | 83.1% |  |
| Female | 19.5% | 16.4% | 14.2% | 17.7% | | 15.5% | 17% | | | 80% | | 87% | | 87% | 16% | | 17% | | 16.2% | | 18.1% | | 15.8% | 15.4% | 16.9% |  |
| **ECOG performance-status score** | | | | | | | | | | | | | | | | | | | | | | | | | |  |
| 0 | 32.2% | 36.4% | 39.2% | 37.0% | | 36.4% | 39% | | | 39% | | 39% | | 39% | 29% | | 27% | | 30.8% | | 33.6% | | 25.8% | 25.9% | 31.7% |  |
| 1 | 67.4% | 63.2% | 60.8% | 62.7% | | 63.6% | 61% | | | 61% | | 61% | | 61% | 71% | | 73% | | 69.2% | | 66.4% | | 74.2% | 74.1% | 68.3% |  |
| **Disease status** | | | | | | | | | | | | | | | | | | | | | | | | | |  |
| Metastatic | 39.4% | 40.0% | 64.2% | 70.5% | | 67.0% | 72% | | | 72% | | 68% | | 67% | / | | / | | 35.0% | | 25.0% | | 7.9% | 9.3% | 14.1% |  |
| Local advanced | 28.2% | 35.8% | 35.3% | 27.6% | | 31.6% | 27% | | | 27% | | 31% | | 32% | / | | / | | 10.3% | | 8.6% | | 92.1% | 90.7% | 85.9% |  |
|  | **CONDOR,n=267** | | | **CheckMate 141,n=361** | | | | | | | **CheckMate 714,n=425** | | | | | | | | |  |  |  |  |  |  |  |
|  | **IG1** | **IG2** | **CG** | **IG1** | **IG2** | | | **CG** | **CG** | | **IG1** | | **IG2** | | | **CG1** | | **CG2** | |  |  |  |  |  |  |  |
|  |  |  |  |  |  |  |  |  |  |  |  |  |  |  |  |  |  |  |  |  |  |  |  |  |  |  |
| **PD-L1 expression level（CPS）** | | | | | | | | | | | | | | | | | | | |  |  |  |  |  |  |  |
| <1 | / | / | / | / | / | | | / | / | | / | | / | | | / | | / | |  |  |  |  |  |  |  |
| ≥1 | / | / | / | / | / | | | / | / | | / | | / | | | / | | / | |  |  |  |  |  |  |  |
| <20 | / | / | / | / | / | | | / | / | | / | | / | | | / | | / | |  |  |  |  |  |  |  |
| ≥20 | / | / | / | / | / | | | / | / | | / | | / | | | / | | / | |  |  |  |  |  |  |  |
| **PD-L1 expression level（TPS）** | | | | | | | | | | | | | | | | | | | |  |  |  |  |  |  |  |
| <1% | / | / | / | 34.0% | 24.7% | | | 27.0% | 38.3% | | 42.1% | | 47.2% | | | 43.9% | | 45.9% | |  |  |  |  |  |  |  |
| ≥1% | 45.9% | 44.8% | 44.8% | 35.4% | 38.7% | | | 54.1% | 44.7% | | 57.9% | | 52.8% | | | 56.1% | | 54.1% | |  |  |  |  |  |  |  |
| <20% | / | / | / | / | / | | | / | / | | / | | / | | | / | | / | |  |  |  |  |  |  |  |
| ≥20% | / | / | / | / | / | | | / | / | | / | | / | | | / | | / | |  |  |  |  |  |  |  |
| **Gender** | | | | | | | | | | | | | | | | | | | |  |  |  |  |  |  |  |
| Male | 85.0% | 80.6% | 79.1% | / | / | | | / | / | | 81.8% | | 85.4% | | | 78.0% | | 77.0% | |  |  |  |  |  |  |  |
| Female | 15.0% | 19.4% | 20.9% | / | / | | | / | / | | 18.2% | | 14.6% | | | 22.0% | | 23.0% | |  |  |  |  |  |  |  |
| **ECOG performance-status score** | | | | | | | | | | | | | | | | | | | |  |  |  |  |  |  |  |
| 0 | 30.1% | 32.8% | 28.4% | 19.7% | 16.2% | | | 21.5% | 23.4% | | 23.9% | | 33.3% | | | 22.0% | | 37.7% | |  |  |  |  |  |  |  |
| 1 | 69.9% | 67.2% | 71.6% | 78.9% | 78.5% | | | 79.7% | 74.5% | | 75.5% | | 66.7% | | | 75.6% | | 60.7% | |  |  |  |  |  |  |  |
| **Disease status** | | | | | | | | | | | | | | | | | | | |  |  |  |  |  |  |  |
| Metastatic | 62.4% | 67.2% | 64.2% | / | / | | | / | / | | 30.8% | | 35.8% | | | 30.5% | | 42.6% | |  |  |  |  |  |  |  |
| Local advanced | 37.6% | 32.8% | 35.8% | / | / | | | / | / | | 39.0% | | 35.0% | | | 43.9% | | 39.3% | |  |  |  |  |  |  |  |

IG,intervention group;CG,control group.

| **Table S5. Incidence of Treatment-related Adverse Events≥3 and Immune-mediated Adverse Events≥3 in Each Therapy** | | | | | | | |
| --- | --- | --- | --- | --- | --- | --- | --- |
| **Events** | **Pem-chemo** | **Nivo-ipi** | **Durva-treme** | **Pembrolizumab** | **Nivolumab** | **Durvalumab** | **Tremelimumab** |
| Treatment-related AEs≥3 | | | | | | | |
| **Anemia** | 0.25 | 0.004 | 0.018 | 0.024 | 0.013 | 0 | 0 |
| **Nausea** | 0.06 | 0 | 0 | 0 | 0 | 0.004 | NR |
| **Vomiting** | 0.04 | 0 | NR | 0.002 | NR | NR | NR |
| **Decrease neutrophil count** | 0.11 | 0.006 | NR | 0.002 | NR | NR | NR |
| **Neutropenia** | 0.18 | 0.006 | 0.004 | 0.002 | NR | 0 | NR |
| **Fatigue** | 0.07 | 0.015 | 0.01 | 0.021 | 0.021 | 0.013 | 0.015 |
| **Asthenia** | 0.03 | 0.006 | 0.021 | 0.007 | 0.004 | 0.003 | 0 |
| **Decreased appetite** | 0.05 | 0.002 | 0.008 | 0.007 | NR | 0 | NR |
| **Decrease platelet count** | 0.05 | 0 | NR | 0.002 | NR | NR | NR |
| **Diarrhea** | 0.03 | 0.017 | 0.016 | 0.009 | NR | 0 | 0.046 |
|  | | | | | | | |
| Immune-mediated AEs≥3 | | | | | | | |
| **Rash** | 0.007 | 0.035 | 0.045 | 0.005 | 0.007 | 0 | 0 |
| **Hypothyroidism** | 0 | 0.003 | 0.122 | 0.002 | 0 | 0 | 0 |
| **Hyperthyroidism** | NR | 0.001 | NR | 0 | 0 | NR | 0 |
| **Immune-mediated lung disease** | NR | 0.012 | NR | 0.017 | 0 | NR | 0 |

NR,not reported.

| **Table S6. Ranking Profiles in the Bayesian Network Meta-analysis（First-line）** | | | | | | | |
| --- | --- | --- | --- | --- | --- | --- | --- |
| **OS** | **Rank Probability** | | | | | | |
| **Treatment plan** | **Rank1st** | **Rank2nd** | **Rank3rd** | **Rank4th** | **Rank5th** | **Rank6th** | **Rank7th** |
| **Nivo** | 64.17% | 16.92% | 13.40% | 3.04% | 1.19% | 0.67% | 0.60% |
| **Pembro-chemo** | 29.25% | 48.81% | 16.64% | 4.10% | 0.82% | 0.28% | 0.11% |
| **Pembro** | 5.83% | 26.19% | 47.79% | 15.20% | 3.32% | 1.20% | 0.48% |
| **Nivo-Ipi** | 0.39% | 5.98% | 15.17% | 46.54% | 16.25% | 9.26% | 6.40% |
| **Durva** | 0.27% | 1.51% | 4.13% | 12.68% | 17.81% | 26.30% | 37.28% |
| **Durva-treme** | 0.08% | 0.57% | 2.28% | 9.12% | 16.72% | 31.69% | 39.53% |
| **SOC** | 0.00% | 0.02% | 0.59% | 9.32% | 43.89% | 30.58% | 15.60% |
| **PFS** | **Rank Probability** | | | | | | |
| **Treatment plan** | **Rank1st** | **Rank2nd** | **Rank3rd** | **Rank4th** | **Rank5th** | **Rank6th** | **Rank7th** |
| **Pembro-chemo** | 69.42% | 16.89% | 8.23% | 4.22% | 1.21% | 0.03% | 0.00% |
| **Nivo** | 13.10% | 12.83% | 13.09% | 19.69% | 26.06% | 10.12% | 5.11% |
| **SOC** | 9.56% | 49.89% | 32.42% | 7.46% | 0.67% | 0.00% | 0.00% |
| **Durva-treme** | 7.89% | 19.61% | 42.45% | 28.94% | 1.06% | 0.05% | 0.00% |
| **Durva** | 0.03% | 0.76% | 3.27% | 36.01% | 49.46% | 8.86% | 1.60% |
| **Pembro** | 0.00% | 0.02% | 0.48% | 2.95% | 14.67% | 41.39% | 40.49% |
| **Nivo-Ipi** | 0.00% | 0.00% | 0.05% | 0.73% | 6.87% | 39.55% | 52.80% |
| **ORR** | **Rank Probability** | | | | | | |
| **Treatment plan** | **Rank1st** | **Rank2nd** | **Rank3rd** | **Rank4th** | **Rank5th** | **Rank6th** | **Rank7th** |
| **Pembro_chemo** | 35.06% | 21.01% | 15.87% | 11.49% | 8.07% | 5.65% | 2.85% |
| **Nivo** | 25.96% | 18.17% | 21.82% | 16.08% | 9.71% | 6.10% | 2.16% |
| **SOC** | 23.45% | 36.64% | 24.64% | 11.05% | 3.43% | 0.69% | 0.09% |
| **Durva_treme** | 5.29% | 6.06% | 9.30% | 15.24% | 20.81% | 24.74% | 18.55% |
| **Pembro** | 4.97% | 8.67% | 13.32% | 19.76% | 19.08% | 16.30% | 17.90% |
| **Durva** | 3.12% | 4.12% | 6.12% | 10.25% | 16.28% | 25.45% | 34.66% |
| **Nivo_Ipi** | 2.15% | 5.33% | 8.93% | 16.13% | 22.62% | 21.07% | 23.78% |
| **AE≥3** | **Rank Probability** | | | | | | |
| **Treatment plan** | **Rank1^st^** | **Rank2^nd^** | **Rank3^rd^** | **Rank4^th^** | **Rank5^th^** | **Rank6^th^** | **Rank7^th^** |
| **Pembro** | 56.42% | 16.19% | 11.56% | 6.90% | 4.85% | 3.06% | 1.02% |
| **Nivo_Ipi** | 14.44% | 30.04% | 21.88% | 16.31% | 9.46% | 4.94% | 2.93% |
| **Durva** | 13.18% | 19.96% | 18.99% | 21.48% | 13.13% | 7.44% | 5.83% |
| **Durva_treme** | 8.32% | 13.94% | 16.23% | 18.64% | 23.97% | 10.05% | 8.85% |
| **Nivo** | 6.51% | 14.83% | 23.10% | 19.75% | 19.36% | 9.31% | 7.14% |
| **Pembro_chemo** | 1.07% | 4.39% | 5.25% | 7.67% | 9.68% | 17.05% | 54.89% |
| **SOC** | 0.07% | 0.65% | 2.99% | 9.25% | 19.55% | 48.15% | 19.35% |

| **Table S7 . Ranking Profiles in the Bayesian Network Meta-analysis(First-line/Selective expression of PD-L1)** | | | | | | |
| --- | --- | --- | --- | --- | --- | --- |
| **OS(PD-L1≥1)** | **Rank Probability** | | | | |  |
| **Treatment plan** | **Rank1^st^** | **Rank2^nd^** | **Rank3^rd^** | **Rank4^th^** | **Rank5^th^** |  |
| **Nivo** | 63.37% | 19.23% | 9.16% | 5.75% | 2.49% |  |
| **Pembro-chemo** | 34.84% | 57.76% | 6.49% | 0.91% | 0.00% |  |
| **Pembro** | 1.42% | 16.37% | 49.00% | 32.39% | 0.81% |  |
| **Nivo-Ipi** | 0.37% | 6.64% | 35.18% | 56.76% | 1.04% |  |
| **SOC** | 0.00% | 0.00% | 0.16% | 4.18% | 95.66% |  |
|  |  | | | | | |
| **PFS(PD-L1≥1)** | **Rank Probability** | | | | |  |
| **Treatment plan** | **Rank1^st^** | **Rank2^nd^** | **Rank3^rd^** | **Rank4^th^** | **Rank5^th^** |  |
| **Pembro-chemo** | 91.76% | 7.53% | 0.65% | 0.06% | 0.00% |  |
| **Nivo** | 6.12% | 13.46% | 17.21% | 17.78% | 45.43% |  |
| **SOC** | 2.07% | 73.56% | 22.59% | 1.74% | 0.04% |  |
| **Nivo-Ipi** | 0.03% | 0.60% | 16.23% | 49.48% | 33.66% |  |
| **Pembro** | 0.02% | 4.85% | 43.32% | 30.94% | 20.88% |  |
|  |  |  |  |  |  |  |
| **OS(PD-L1≥20)** | **Rank Probability** | | | | | |
| **Treatment plan** | **Rank1^st^** | **Rank2^nd^** | **Rank3^rd^** | **Rank4^th^** | **Rank5^th^** | **Rank6^th^** |
| **Pembro-chemo** | 49.65% | 39.71% | 9.64% | 0.89% | 0.10% | 0.01% |
| **Pembro** | 43.25% | 43.60% | 11.75% | 1.22% | 0.15% | 0.03% |
| **Nivo-ipi** | 6.66% | 14.48% | 55.26% | 14.17% | 5.49% | 3.94% |
| **Durva** | 0.41% | 1.93% | 16.02% | 35.52% | 22.45% | 23.67% |
| **SOC** | 0.00% | 0.00% | 2.33% | 29.86% | 45.37% | 22.44% |
| **Durva-treme** | 0.03% | 0.28% | 4.99% | 18.34% | 26.45% | 49.91% |
| **PFS(PD-L1≥20)** | **Rank Probability** | | | | | |
| **Treatment plan** | **Rank1^st^** | **Rank2^nd^** | **Rank3^rd^** | **Rank4^th^** | **Rank5^th^** | **Rank6^th^** |
| **Pembro-chemo** | 91.42% | 6.45% | 1.13% | 0.48% | 0.32% | 0.20% |
| **Pembro** | 3.56% | 36.83% | 16.64% | 12.01% | 10.32% | 20.65% |
| **SOC** | 0.44% | 19.44% | 40.47% | 27.49% | 9.33% | 2.82% |
| **Nivo-ipi** | 4.00% | 26.28% | 17.24% | 13.58% | 10.97% | 27.93% |
| **Durva** | 0.45% | 7.92% | 14.30% | 23.40% | 31.68% | 22.25% |
| **Durva-treme** | 0.14% | 3.08% | 10.21% | 23.03% | 37.38% | 26.16% |
|  |  |  |  |  |  |  |

| **Table S8. Ranking Profiles in the Bayesian Network Meta-analysis(Second-line)** | | | | | | |
| --- | --- | --- | --- | --- | --- | --- |
| **OS** | **Rank Probability** | | | | | |
| **Treatment plan** | **Rank1^st^** | **Rank2^nd^** | **Rank3^rd^** | **Rank4^th^** | **Rank5^th^** | **Rank6^th^** |
| **Nivo** | 89.91% | 8.20% | 1.58% | 0.26% | 0.04% | 0.00% |
| **Pembro** | 7.63% | 64.69% | 21.60% | 4.89% | 1.10% | 0.09% |
| **Durva** | 2.34% | 24.99% | 52.92% | 15.20% | 4.29% | 0.27% |
| **SOC** | 0.00% | 0.22% | 13.20% | 45.36% | 37.71% | 3.51% |
| **Durva-trerne** | 0.08% | 1.58% | 9.90% | 32.80% | 53.85% | 1.80% |
| **Treme** | 0.05% | 0.33% | 0.81% | 1.49% | 3.00% | 94.32% |
| **PFS** | **Rank Probability** | | | | | |
| **Treatment plan** | **Rank1^st^** | **Rank2^nd^** | **Rank3^rd^** | **Rank4^th^** | **Rank5^th^** | **Rank6^th^** |
| **Nivo** | 75.10% | 11.20% | 6.05% | 4.35% | 2.97% | 0.33% |
| **SOC** | 4.55% | 35.50% | 37.68% | 17.97% | 4.08% | 0.23% |
| **Durva** | 12.12% | 27.39% | 21.72% | 28.03% | 10.09% | 0.65% |
| **Pembro** | 6.47% | 19.19% | 22.12% | 24.34% | 25.00% | 2.87% |
| **Durva-treme** | 1.53% | 6.21% | 11.79% | 24.20% | 54.73% | 1.54% |
| **Treme** | 0.22% | 0.51% | 0.65% | 1.11% | 3.13% | 94.38% |
| **ORR** | **Rank Probability** | | | | | |
| **Treatment plan** | **Rank1^st^** | **Rank2^nd^** | **Rank3^rd^** | **Rank4^th^** | **Rank5^th^** | **Rank6^th^** |
| **Nivo** | 76.94% | 9.71% | 5.53% | 3.46% | 3.42% | 0.95% |
| **Pembro** | 8.99% | 36.86% | 18.88% | 16.50% | 16.22% | 2.56% |
| **SOC** | 0.97% | 11.21% | 29.64% | 30.41% | 25.72% | 2.05% |
| **Durva** | 7.04% | 22.94% | 22.80% | 23.83% | 22.27% | 1.12% |
| **Durva-treme** | 5.52% | 18.34% | 21.77% | 23.88% | 29.21% | 1.28% |
| **Treme** | 0.54% | 0.94% | 1.38% | 1.92% | 3.17% | 92.05% |
| **AE≥3** | **Rank Probability** | | | | | |
| **Treatment plan** | **Rank1^st^** | **Rank2^nd^** | **Rank3^rd^** | **Rank4^th^** | **Rank5^th^** | **Rank6^th^** |
| **Nivo** | 37.15% | 25.88% | 17.76% | 9.73% | 7.45% | 2.04% |
| **Pembro** | 33.73% | 33.24% | 18.18% | 8.99% | 5.24% | 0.62% |
| **Durva** | 19.63% | 26.73% | 36.80% | 12.27% | 3.75% | 0.82% |
| **Durva-treme** | 1.56% | 4.99% | 12.41% | 39.36% | 36.24% | 5.44% |
| **Treme** | 7.91% | 8.86% | 12.62% | 23.73% | 28.89% | 17.99% |
| **SOC** | 0.02% | 0.30% | 2.24% | 5.92% | 18.43% | 73.09% |

| **Table S9. Ranking Profiles in the Bayesian Network Meta-analysis(Second-line/Selective expression of PD-L1)** | | | |
| --- | --- | --- | --- |
| **OS(PD-L1≥1)** | **Rank Probability** | | |
| **Treatment plan** | **Rank1^st^** | **Rank2^nd^** | **Rank3^rd^** |
| **Nivo** | 91.76% | 8.21% | 0.04% |
| **Pembro** | 8.24% | 91.16% | 0.59% |
| **SOC** | 0.00% | 0.63% | 99.37% |
| **PFS(PD-L1≥1)** | **Rank Probability (%)** | | |
| **Treatment plan** | **Rank1^st^** | **Rank2^nd^** | **Rank3^rd^** |
| **Nivo** | 98.96% | 0.85% | 0.19% |
| **Pembro** | 1.02% | 91.40% | 7.58% |
| **SOC** | 0.02% | 7.75% | 92.23% |


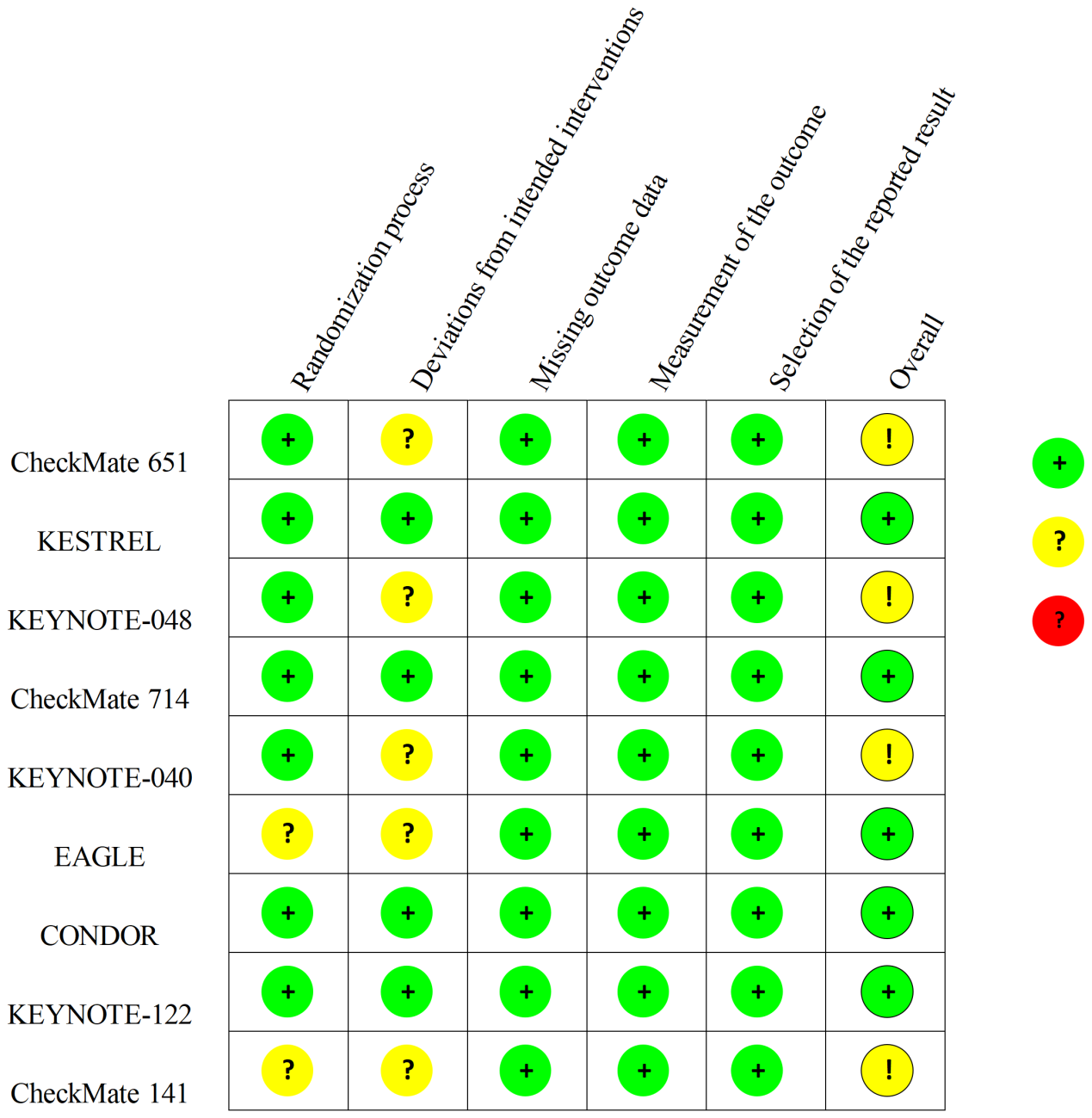


**Figure S1. Summary of results from assessment of studies by using Cochrane Risk of Bias Tool 2.0**

Studies were classified into one of three categories: low, high risk or having “some concerns”


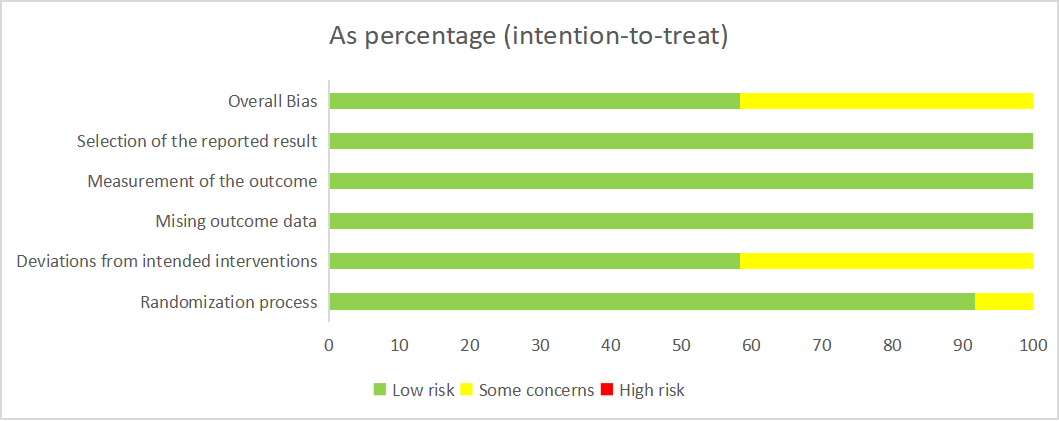


**Figure S2. Graph of results from assessment of studies by using Cochrane Risk of Bias Tool 2.0**


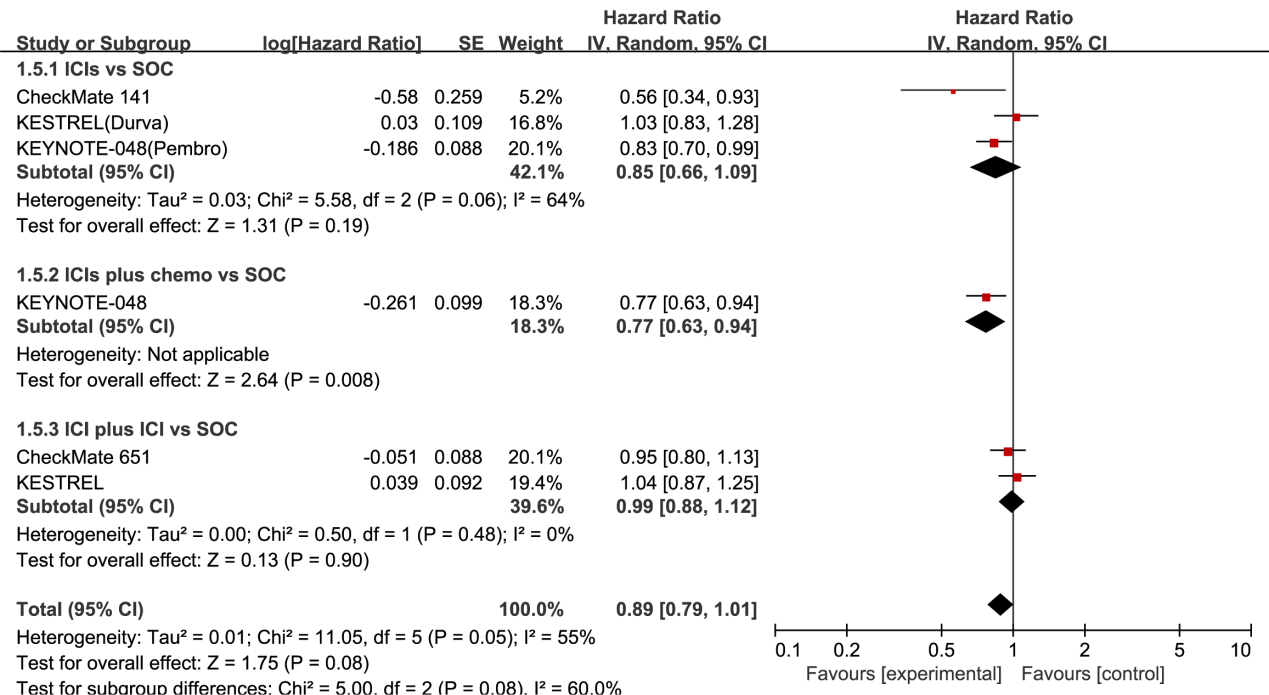


**Figure S3. Forest Plot Comparing First-line Immunotherapy vs. SOC in Patients with R/M**

**HNSCC Using OS as the Endpoint**

**
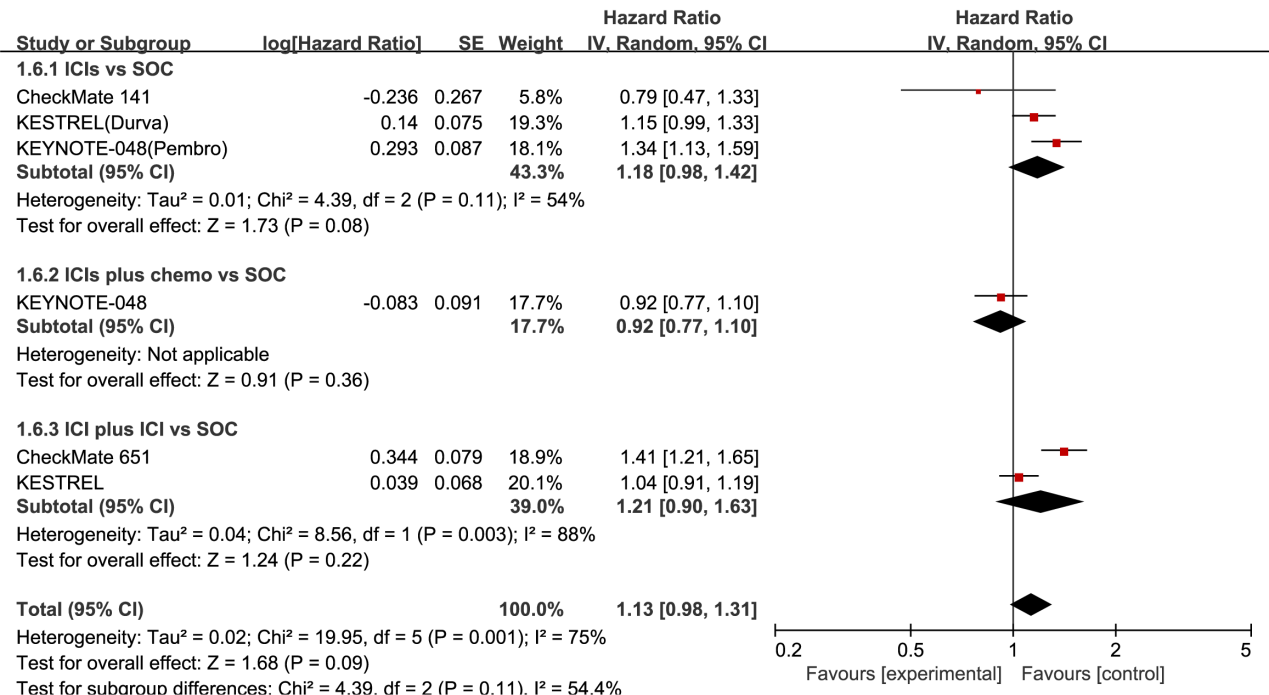
**

**Figure S4. Forest Plot Comparing First-line Immunotherapy vs. SOC in Patients with R/M**

**HNSCC Using PFS as the Endpoint**

**
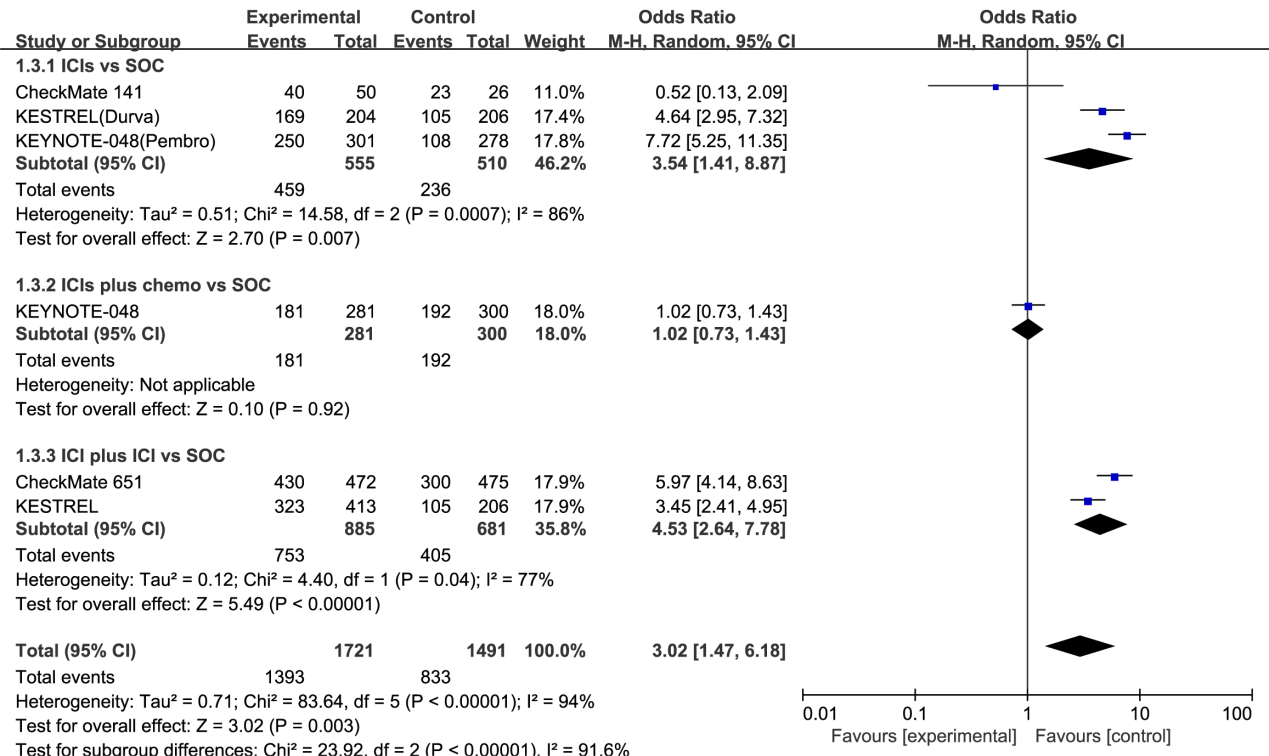
**

**Figure S5. Forest Plot Comparing First-line Immunotherapy vs. SOC in Patients with R/M**

**HNSCC Using ORR as the Endpoint**

**
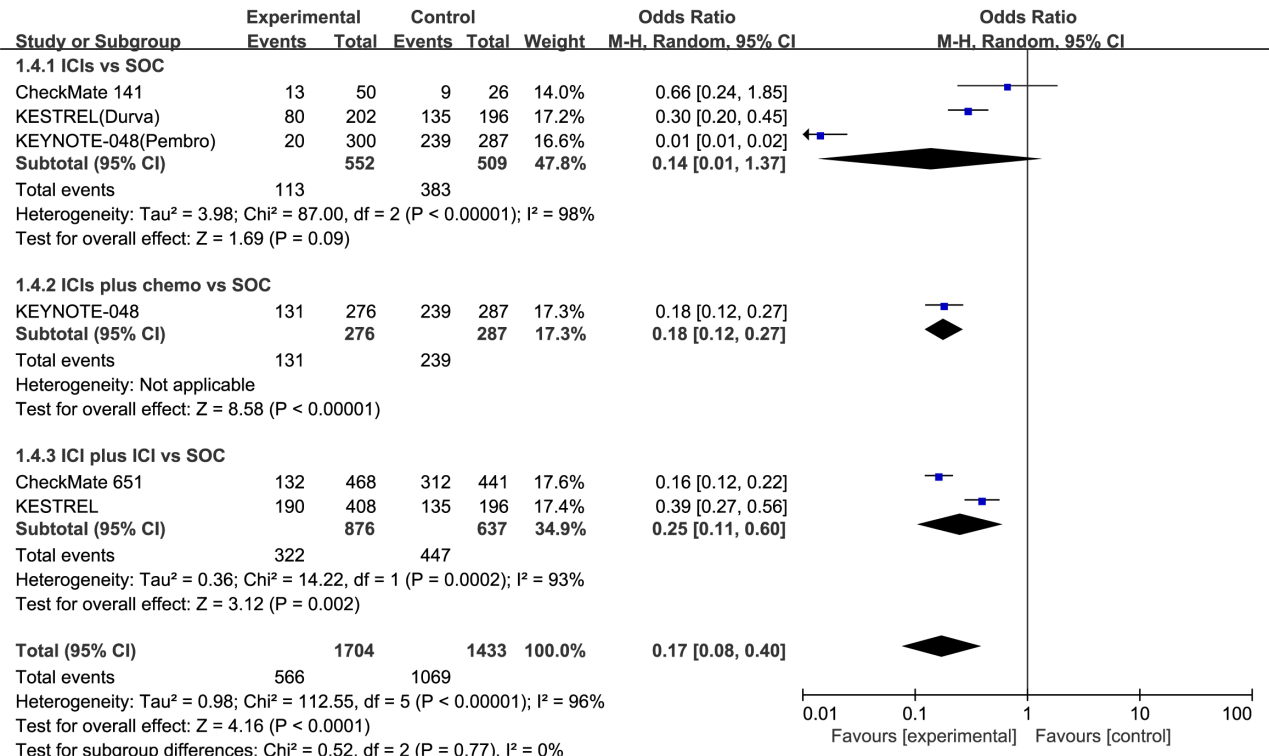
**

**Figure S6. Forest Plot Comparing First-line Immunotherapy vs. SOC in Patients with R/M**

**HNSCC Using AE≥3 as the Endpoint**

**
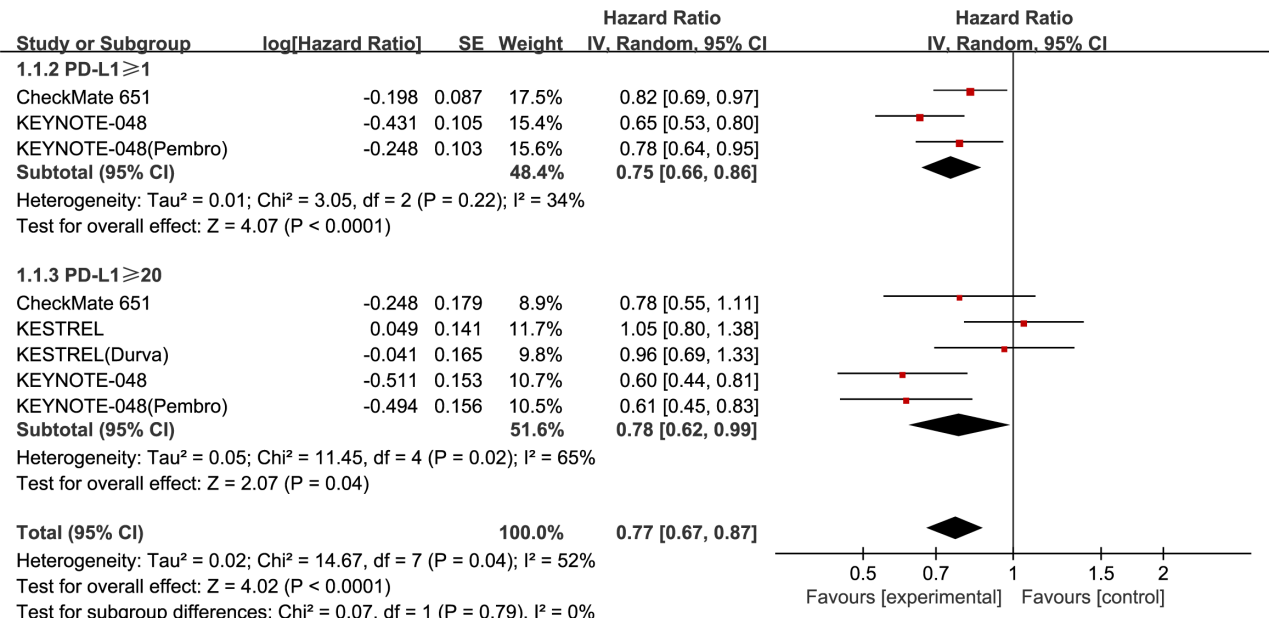
**

**Figure S7. Forest Plot Comparing First-line Immunotherapy vs. SOC in Patients with R/M HNSCC Grouped by PD-L1 Expression Levels Using OS as the Endpoint**

**
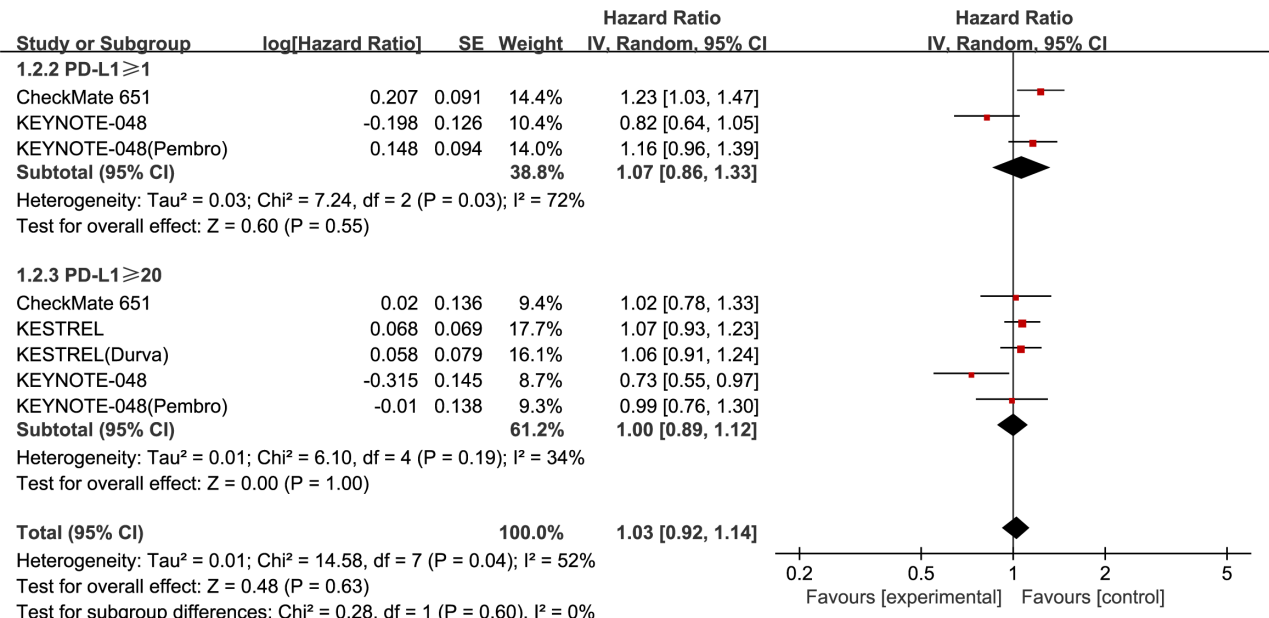
**

**Figure S8. Forest Plot Comparing First-line Immunotherapy vs. SOC in Patients with R/M HNSCC Grouped by PD-L1 Expression Levels Using PFS as the Endpoint**

**
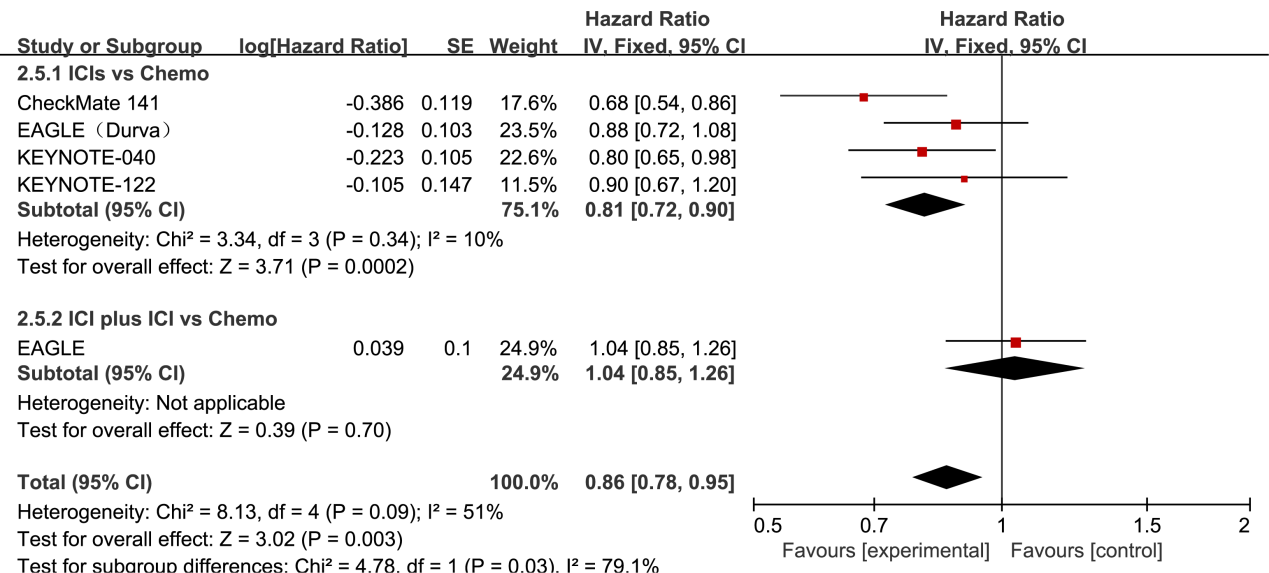
**

**Figure S9. Forest Plot Comparing Second-line Immunotherapy vs. SOC in Patients with R/M HNSCC Using OS as the Endpoint**

**
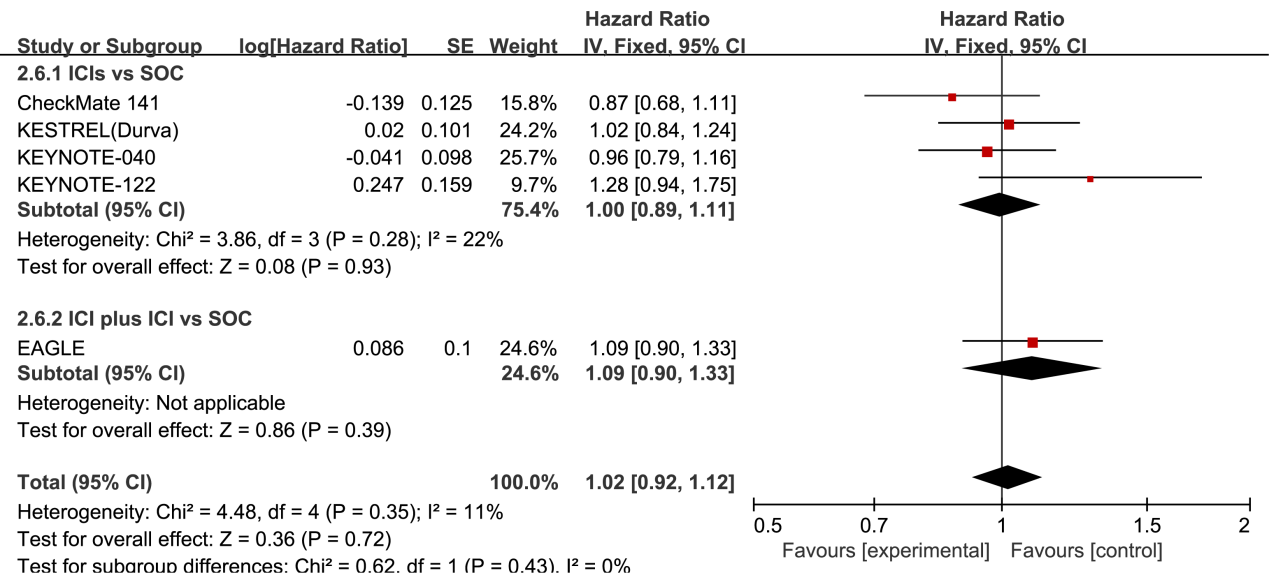
**

**Figure S10. Forest Plot Comparing Second-line Immunotherapy vs. SOC in Patients with R/M HNSCC Using PFS as the Endpoint**

**
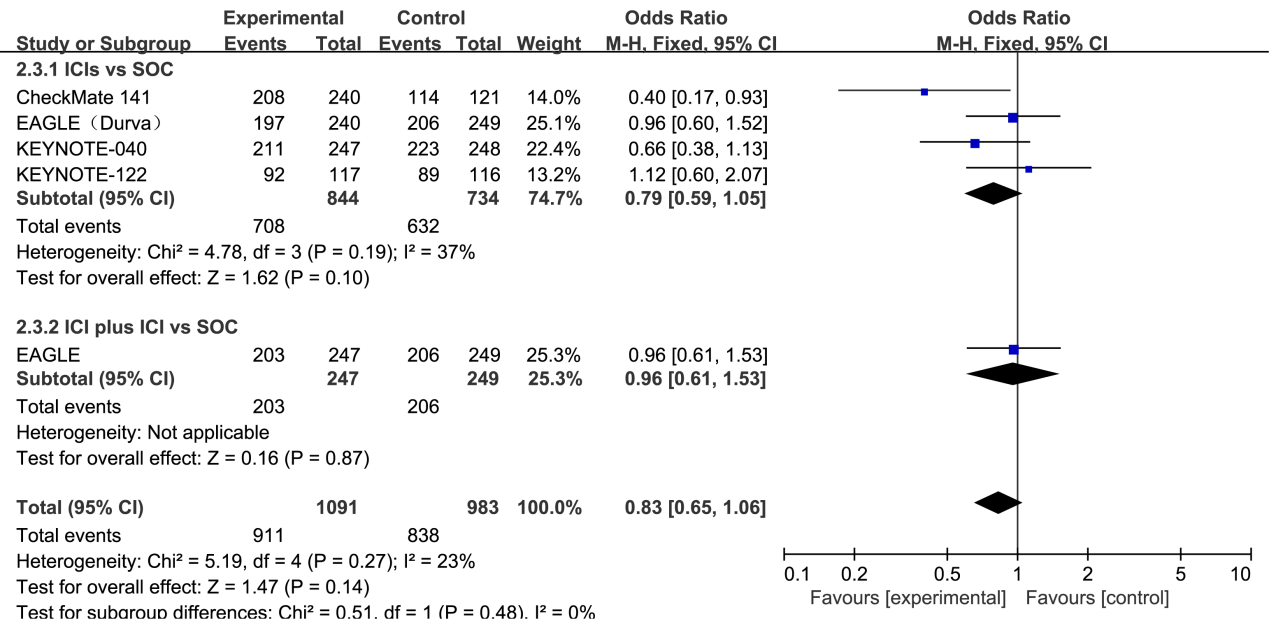
**

**Figure S11. Forest Plot Comparing Second-line Immunotherapy vs. SOC in Patients with R/M HNSCC Using ORR as the Endpoint**

**
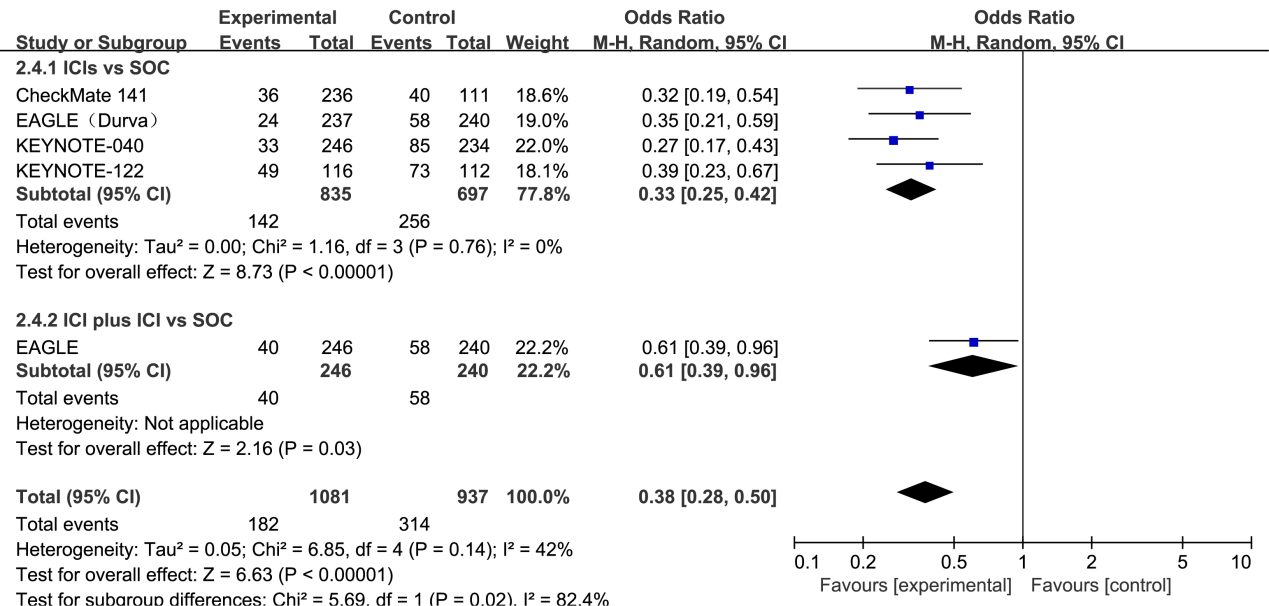
**

**Figure S12. Forest Plot Comparing Second-line Immunotherapy vs. SOC in Patients with R/M HNSCC Using AE≥3 as the Endpoint**

**
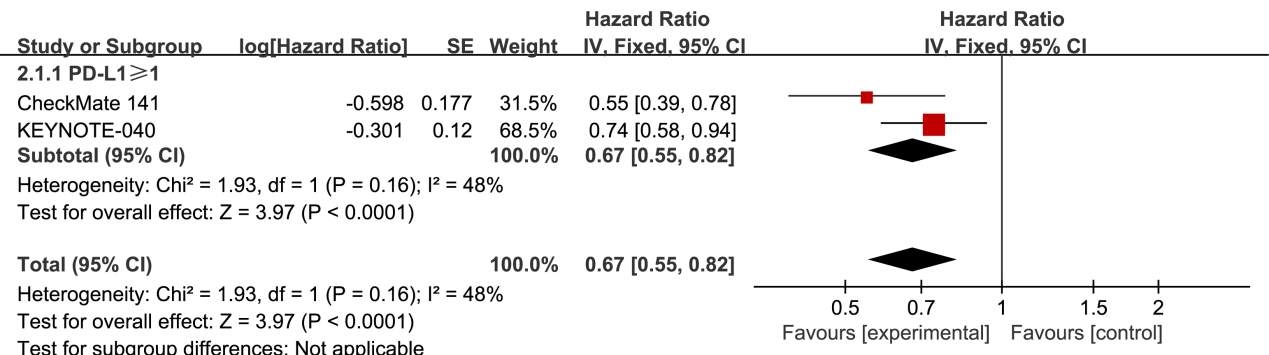
**

**Figure S13. Forest Plot Comparing Second-line Immunotherapy vs. SOC in Patients with R/M HNSCC and PD-L1 Expression ≥1% Using OS as the Endpoint**

**
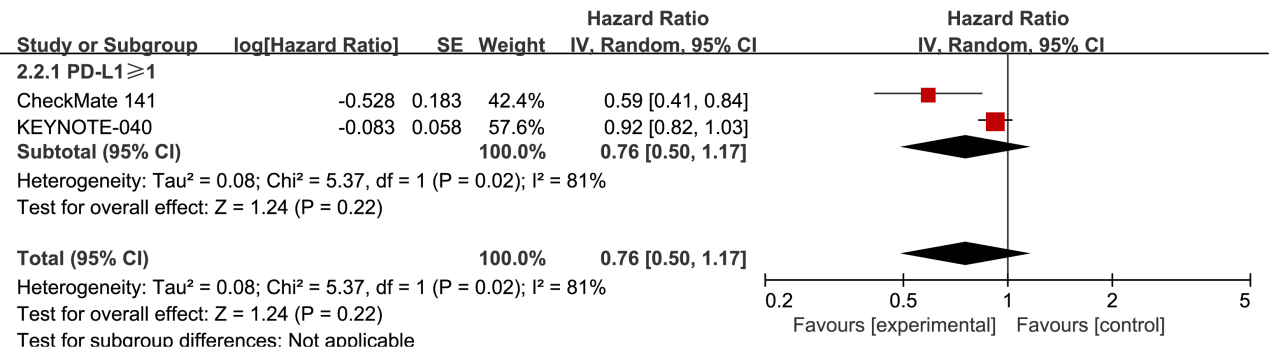
**

**Figure S14. Forest Plot Comparing Second-line Immunotherapy vs. SOC in Patients with R/M HNSCC and PD-L1 Expression ≥1% Using PFS as the Endpoint**

**
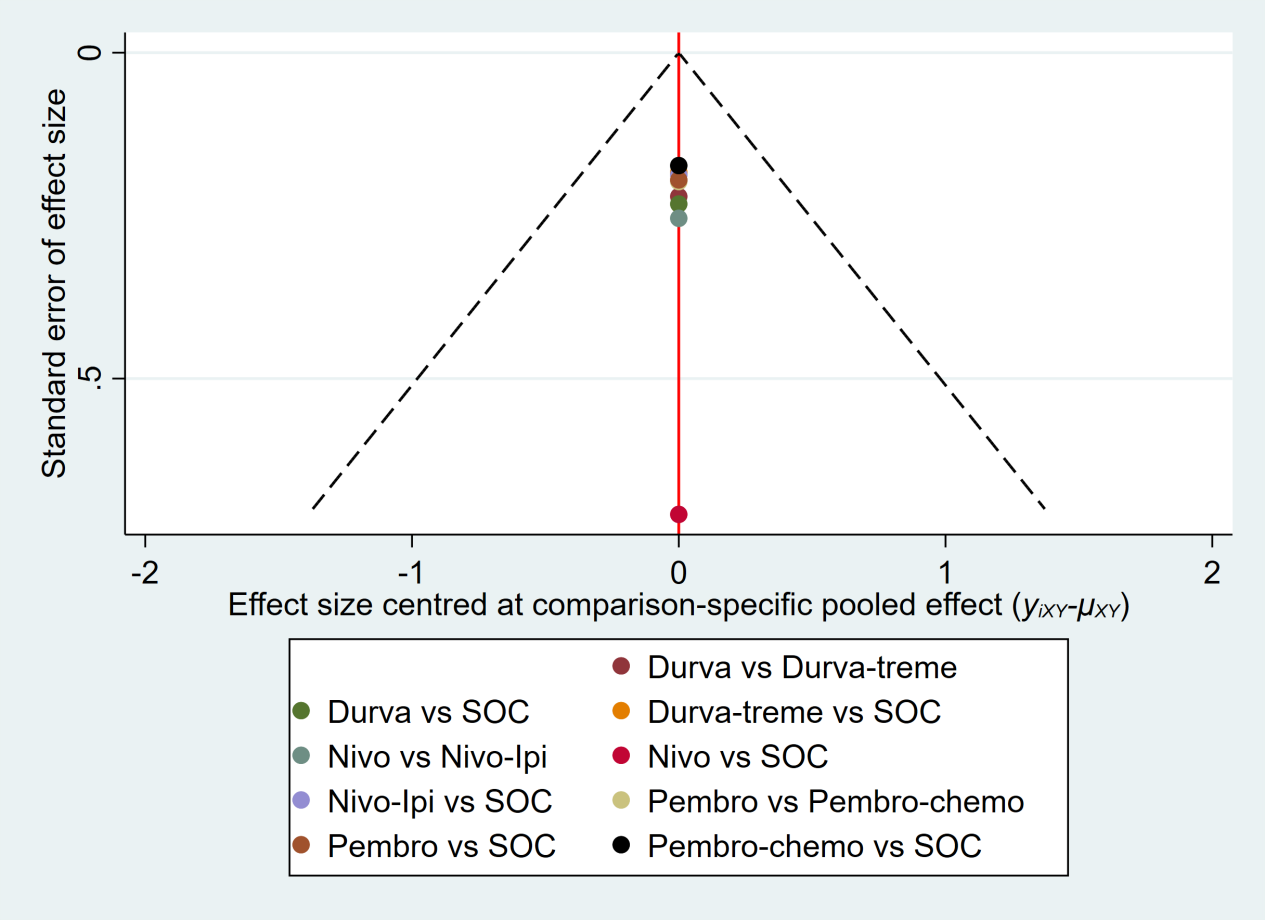
**

**Figure S15. Funnel Plot of Network Meta-analysis of First-line Immunotherapy in Patients with R/M HNSCC Using OS as the Endpoint**

**
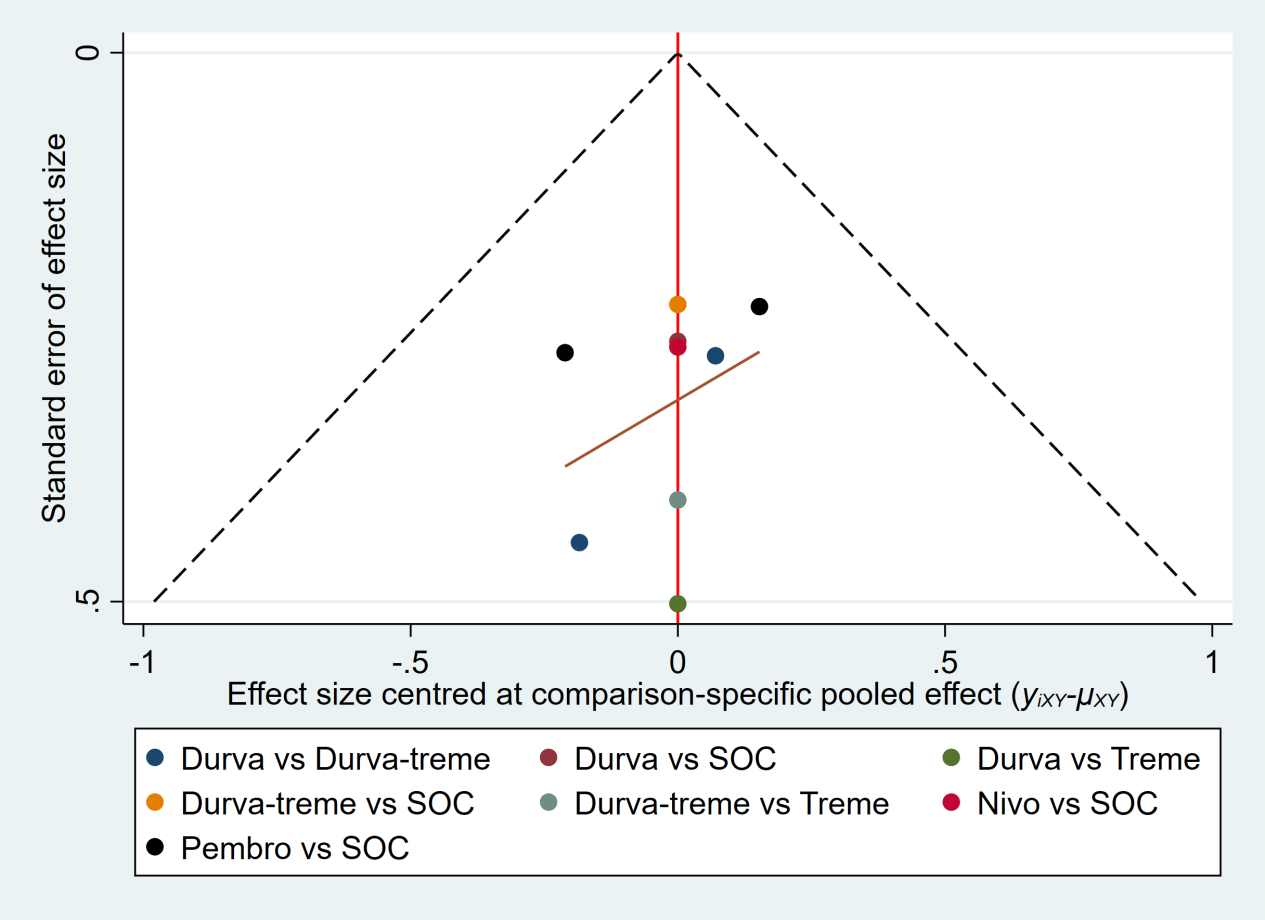
**

**Figure S16. Funnel Plot of Network Meta-analysis of Second-line Immunotherapy in Patients with R/M HNSCC Using OS as the Endpoint**
